# Novel Viroid‐Like RNAs Naturally Infect a Filamentous Fungus

**DOI:** 10.1002/advs.202204308

**Published:** 2022-12-14

**Authors:** Kaili Dong, Chuan Xu, Ioly Kotta‐Loizou, Jingjing Jiang, Ruiying Lv, Linghong Kong, Shifang Li, Ni Hong, Guoping Wang, Robert H. A. Coutts, Wenxing Xu

**Affiliations:** ^1^ Hubei Hongshan Laboratory Wuhan Hubei 430070 P. R. China; ^2^ Key Laboratory of Horticultural Crop (Fruit Trees) Biology and Germplasm Creation of the Ministry of Agriculture Wuhan Hubei 430070 P. R. China; ^3^ Key Lab of Plant Pathology of Hubei Province Wuhan Hubei 430070 P. R. China; ^4^ College of Plant Science and Technology Huazhong Agricultural University Wuhan Hubei 430070 P. R. China; ^5^ Department of Life Sciences Faculty of Natural Sciences Imperial College London London SW7 2AZ UK; ^6^ Department of Clinical Pharmaceutical and Biological Science School of Life and Medical Sciences University of Hertfordshire Hatfield AL10 9AB UK; ^7^ Environment and Plant Protection Institute Chinese Academy of Tropical Agricultural Sciences Xueyuan Road, Longhua District Haikou Hainan 571101 P. R. China; ^8^ State Key Laboratory for Biology of Plant Diseases and Insect Pests Institute of Plant Protection Chinese Academy of Agricultural Sciences Beijing 100193 P. R. China

**Keywords:** circular RNA, fungus, mycoviroid, viroid, viroid‐like

## Abstract

To date, viroids have been found to naturally infect only plants, resulting in substantial losses for some crops. Whether viroids or viroid‐like RNAs naturally infect non‐plant hosts remains unknown. Here the existence of a set of exogenous, single‐stranded circular RNAs, ranging in size from 157 to 450 nucleotides, isolated from the fungus *Botryosphaeria dothidea* and nominated *B. dothidea* RNAs (BdcRNAs) is reported. BdcRNAs replicate autonomously in the nucleus via a rolling‐circle mechanism following a symmetric pathway. BdcRNA infection induces symptoms, because BdcRNAs can apparently modulate, to different degrees, specific biological traits (e.g., alter morphology, decrease growth rate, attenuate virulence, and increase or decrease tolerance to osmotic and oxidative stress) of the host fungus. Overall, BdcRNAs have genome characteristics similar to those of viroids and exhibit pathogenic effects on fungal hosts. It is proposed that these novel fungus infecting RNAs should be termed mycoviroids. BdcRNA(s) may be considered additional inhabitants at the frontier of life in terms of genomic complexity, and represent a new class of acellular entities endowed with regulatory functions, and novel epigenomic carriers of biological information.

## Graphical abstract

A novel class of viroid‐like RNAs, termed as mycoviroids, naturally infects a filamentous fungus *Botryosphaeria dothidea* responsible for fungal attenuated virulence and growth rate. These mycoviroids range in size between 157 and 450 nucleotides, replicate autonomously in the nucleus via a rolling‐circle replication mechanism following symmetric pathways, and regulate the metabolic pathways of the host fungus.

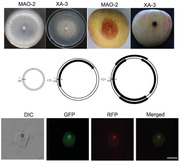

## 1 Introduction

Viroids are small pathogenic single‐stranded (ss) non‐protein‐coding RNAs varying in size from 246 to 401 nucleotides (nts), which replicate autonomously following inoculation of higher plants.^[^ ^1^ ^]^ Viroids are classified into two families, *Pospiviroidae* and *Avsunviroidae*, based on their RNA secondary structure, ribozyme activity, subcellular location, genome organization, replication, and phylogenetic relationships.^[^ ^1^ ^]^ Members of the *Pospiviroidae* family adopt a rod‐like secondary structure with five structural/functional domains, including a central conserved region (CCR), and are localized and replicated in the nucleus.^[^ ^1b^ ^]^ Members of the *Avsunviroidae* family adopt a branched or quasi‐rod‐like secondary structure, lack a CCR, form hammerhead ribozymes (HHRz), and are localized and replicate in chloroplasts.^[^ ^1^ ^]^ Viroids replicate via a rolling circle mechanism in three steps, RNA transcription, processing, and ligation; they follow either an asymmetric pathway catalyzed by host enzymes (RNA polymerase II, RNase I, and DNA ligase I) for members of the *Pospiviroidae*, or a symmetric pathway for members of the *Avsunviroidae* that are self‐cleaved by HHRz, which functionally substitutes for RNase III during replication.^[^ ^1a^ ^]^ With a minimal genome size and simple structure, viroids are giants in terms of functional versatility in the RNA world. Viroids can override cellular RNA degradation machinery and enlist host‐encoded factors for self‐replication in specific subcellular compartments and for trafficking through plants.^[^ ^2^ ^]^

Viroids infect horticultural plants, including vegetables, fruits, and ornamentals, and cause devastating diseases in some crops.^[^ ^1b^ ^]^ To date, viroids have been found to naturally infect only plants, although some studies have arguably suggested that they can also replicate in yeast, filamentous fungi, oomycetes, and cyanobacteria following artificial inoculation.^[^ ^3^ ^]^ Whether viroids or viroid‐like RNAs naturally infect other organisms apart from plants is not known. If they do, their molecular and biological traits, phylogenetic relationships with viroids that infect plants, and interactions with their host are of great interest.

Phytopathogenic fungi cause numerous diseases in plants resulting in significant, often catastrophic, annual economic crop losses, and have led to human famine throughout history. Some fungi play crucial roles in ecosystems and nutrient recycling as decomposers of dead plant material. Fungi are also used in the food industry and for antibiotic production.^[^ ^4^ ^]^ Other fungi cause animal and human diseases often resulting in death and disability.^[^ ^5^ ^]^ Fungi connect diverse life kingdoms and accommodate diverse microbe types: they can be parasites of plants, animals (including humans), and other fungi, whilst also harboring viruses (known as mycoviruses or fungal viruses), protists, bacteria, and prions. Fungi together with these microbes play important roles in influencing other life kingdoms; therefore understanding, controlling, and applying fungal communities with the aid of symbiotic organisms attract major theoretical and practical interest.

*Botryosphaeria dothidea* (Moug.: Fr.) Cesati & De Notaris (anamorph *Fusicoccum aesculi* Corda) is an important phytopathogenic fungus with a worldwide distribution, which infects numerous plant species including apple, pear, and grape causing symptoms that include die‐back, stem and shoot blight, gummosis, canker and fruit rot.^[^ ^6^ ^]^ It is known that most fungal species (including *B. dothidea*) are highly divergent in their biological traits including morphology, growth rate, and virulence in the same host; even if their genetic background is identical, as is the case for conidium‐ or protoplast generated sub‐isolates of the same parental strain. These changes, which are beyond our understanding, based on current genetic knowledge, might be elicited by certain as yet unknown regulatory factors or biological agents.

Here, we have characterized a set of exogenous circular RNAs (ecRNAs) isolated from an attenuated phytopathogenic strain of *B. dothidea* and found that they display molecular and biological features similar to those of viroids, and modulate to different degrees specific biological traits (e.g., growth rate, morphology, and virulence) of the host fungus by regulating related metabolism pathways. Our experimental data indicate that these ecRNAs are viroid‐like RNAs that induce symptomatic infections in their fungal hosts.

## 2 Results

### 2.1 A Phytopathogenic Fungus Harbors a Complex Pattern of dsRNAs and Circular RNAs

**Figure 1:**
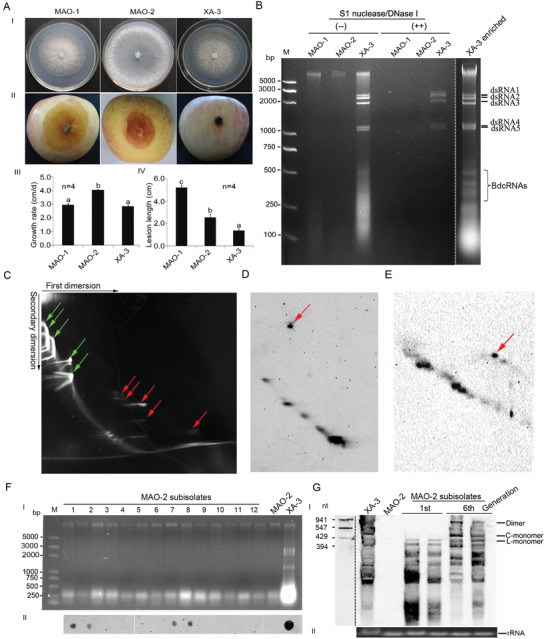
Morphology and virulence of *B. dothidea* strains and characterization of their associated nucleic acids. A) I) Colony morphology, II) virulence on apple (*M. domestica* cv. “Fuji”), III) histograms of growth rates (*n* = 3), and IV) lesion lengths on apple (*n* = 4) of strains MAO‐1, MAO‐2, and XA‐3, respectively. Bars in each histogram labeled with the same letters are not significantly different (*P* > 0.05) according to the least significant difference test (1‐way ANOVA); and error bars represent standard deviation (SD). B) Electrophoretic analysis on a 2.0% agarose gel of nucleic acid preparations from strains MAO‐1, MAO‐2, and XA‐3 before (−) and after (++) digestion with both S1 nuclease and DNase I; *B. dothidea* circular RNAs (BdcRNAs) enriched from strain XA‐3 without enzyme treatment (right panel). The dsRNAs 1–5 represent the genome of *B. dothidea* RNA virus 1 (BdRV1). C) 2D polyacrylamide gel electrophoresis (2D‐PAGE) analysis of nucleic acid preparations from strain XA‐3 as shown in panel B. The green arrows indicate BdRV1 dsRNAs 1–5, followed by consecutive and traced host RNAs, and the red arrows indicate BdcRNAs, as judged by the corresponding electrophoretic positions and numbers. D,E) Northern blotting analysis of nucleic acid preparation from strain XA‐3 after 2D‐PAGE analysis of BdcRNAs 1 and 2, respectively. Both 2D‐PAGE analyses are independent on this as shown in panel C. The signals refer to the circular (indicated by red arrows) and linear forms of BdcRNA 1 or 2. F) I) Nucleic acids extracted from protoplast‐generated sub‐isolates together with strains MAO‐2 and XA‐3 and II) corresponding dot blotting analysis of BdcRNA 2. G) Northern blotting analysis of BdcRNA 2 in the first and sixth generations of two positive sub‐isolates chosen randomly, using transcribed BdcRNA 2 fragments as the marker. I) C‐ and L‐monomer refer to the circular and linear forms, respectively. II) The aliquots of the extracted fungal nucleic acids were analyzed on 1% agarose gel, stained by ethidium bromide, and involved as a loading control with the rRNA bands.

Three *B. dothidea* strains, XA‐3, MAO‐1, and MAO‐2, with differing morphology and virulence, were isolated from apple branches (*Malus domestica* Borkh. cv. “Fuji”). Strain XA‐3 was avirulent or weakly virulent on apple or pear fruits eliciting lesions < 1.4 cm in diameter as compared to those caused by the MAO‐1 and MAO‐2 strains which were >3.0 cm in diameter (**Figure** **1**A, panel II and IV). Strain XA‐3 has a morphology and growth rate similar to strain MAO‐1 on potato dextrose agar (PDA; Figure 1A, panel I and III). Following culture on PDA colonies of both the XA‐3 and MAO‐1 strains were initially white but subsequently turned grey, with a flat surface and dense cotton‐like aerial mycelium. In contrast, strain MAO‐2 had an increased growth rate of 4.0 mm per day and formed white colonies with thin mycelia collapsed at the center (Figure 1AI,III).

To investigate whether the presence of biological agents such as viruses was responsible for the divergent morphology and/or virulence of strain XA‐3, nucleic acid preparations enriched in dsRNA were obtained from XA‐3 mycelia and analyzed by agarose gel electrophoresis. A complex pattern of dsRNA bands was detected, including a population of larger elements, ranging from 1 to 2.4 kbp in size, and smaller elements, ranging from 100 to 500 bp in size (Figure 1B). These elements were detected exclusively in the XA‐3 strain and were not present in the MAO‐1 or MAO‐2 strains (Figure 1B). The sequences of the full‐length complementary DNAs (cDNAs) of the dsRNAs were determined by assembling partial cDNA sequences amplified from each individually purified dsRNA using RT‐PCR with tagged random primers and rapid amplification of cDNA ends (RACE). The assembled sequences of dsRNAs 1–5 were respectively 2399, 2188, 1965, 1131, and 1059 bp in size and were deposited in GenBank under accession numbers KT372135‐KT372139. Following BLASTn searches of public databases, the dsRNAs sequenced in this study were found to be 99.0–100% identical to those of *B. dothidea* RNA virus 1 (BdRV1) dsRNAs 1–5,^[^ ^7^ ^]^ constituting a strain of this virus. However, using the same experimental approach, we failed to determine the terminal sequences of any of the smaller RNAs, leading us to conclude that they lacked accessible free 3′ and 5′ termini and, consequently, might have a circular structure. We then obtained preparations enriched in these RNAs from strain XA‐3, as previously described for circular viroid RNAs^[^ ^8^ ^]^ (last lane in Figure 1B), and subjected them to native 2D polyacrylamide gel electrophoresis (2D‐PAGE)^[^ ^9^ ^]^ prior to further fractionation using fully denaturing conditions in the second dimension. Several RNA species were detected in the second denaturing gel, which migrated slower than host RNAs (Figure 1C indicated by red arrows). Such behavior is consistent with the presence of circular RNAs, since all linear RNAs from a crude extract ran on the diagonal‐like front, while the circular forms migrated slower and were separated behind all other nucleic acids following 2D‐PAGE.^[^ ^9^ ^]^ By contrast, the BdRV1 dsRNAs migrated together with host nucleic acids (Figure 1C, indicated by green arrows). These results suggest that the small RNA bands visualized on gels are most likely circular RNAs and were accordingly nominated *B. dothidea* circular RNAs (BdcRNAs).

### 2.2 Full Length Sequences and Strand Polarity of the BdcRNAs

To obtain full‐length sequences of each BdcRNA abutted primer pairs of opposite polarity were designed (Table S1, Supporting Information) based on the assembled contigs of partial cDNAs, amplified using RT‐PCR with tagged random primers from each individually purified BdcRNA. Six amplified cDNA bands, corresponding to RNAs ≈100–500 bp long were obtained (with primer pairs F1/R1, Figure S1A, Supporting Information). Furthermore, RT‐PCR amplification using additional adjacent primers (F2/R2) of opposite polarity based on Sanger derived sequences obtained for BdcRNAs 1, 2.1, and 3.1 (Table S1, Supporting Information), produced cDNA bands of the expected sizes, further supporting their circular nature (Figure S1A, Supporting Information). Sequence analysis of the full‐length cDNAs from the six BdcRNAs indicated that they were respectively 450, 429, 396, 374, 221, and 157 nt in length and were designated as BdcRNAs 1, 2 (2.1, 2.2, and 2.3) and 3 (3.1 and 3.2) based on sequence similarity.

To confirm further the circular nature of the BdcRNAs, complementary strands of BdcRNAs 1 and 2.1 were DIG‐labeled by in vitro transcription of their corresponding full‐length cDNAs and used as northern blotting probes for total nucleic acid extracts from strain XA‐3 following fractionation by 2D‐PAGE (Figure 1D). Using the DIG‐labeled BdcRNA 1 antisense riboprobe, a single dot hybridization signal was observed migrating behind the remaining signals in support of the notion that one of the slowly migrating nucleic acid bands observed following 2D‐PAGE corresponds to the circular form of BdcRNA 1, while those in the diagonal‐like front correspond to linear intermediates (Figure 1D). When a DIG‐labeled antisense BdcRNA 2.1 riboprobe was used in further northern analysis following 2D‐PAGE a similar result was obtained (Figure 1E). These results with BdcRNAs 1 and 2 as representatives confirmed the circular nature of the BdcRNAs.

To confirm the infectivity of the BdcRNAs independent of BdRV1 or other potential co‐infecting mycoviruses, BdcRNAs co‐migrating with 200–500 bp markers were eluted from agarose gels and used to transfect strain MAO‐2. Twelve protoplast‐ derived colonies were picked at random and transferred to new PDA plates to generate fresh mycelia and avoid contamination with inoculum BdcRNA. Following the development of fresh mycelia, 0.5 cm diameter discs were excised from each colony margin and sub‐cultured on cellophane membrane covered PDA prior to nucleic acid extraction using silica spin columns to enrich dsRNA concentration.^[^ ^10^ ^]^ These extracts, which were devoid of any BdRV1 dsRNAs (Figure 1FI), were subjected to dot blotting hybridization using a DIG‐labeled BdcRNA 2.1 antisense riboprobe. This analysis revealed that 4/12 protoplast‐ derived sub‐isolates were infected with BdcRNA 2.1 (Figure 1FII). Sub‐cultures of up to six generations of 2/4 infected sub‐isolates above were processed and probed revealing that the BdcRNA 2 band patterns were similar to those of strain XA‐3, but at higher titers with additional RNA molecules (including dimeric and circular forms) as compared to the original transfectants (Figure 1G). Following RT‐PCR amplification it was shown that all transfectants were free of BdRV1 but were infected with BdcRNA 2 (Figure S1B, Supporting Information). These results strongly support the notion that BdcRNAs are infectious and can initiate replication following protoplast transfection.

The positive‐sense strands of the BdcRNAs were defined as those that accumulated to higher titers as compared to their counterparts in strain XA‐3 mycelia, as illustrated by dot blotting with the corresponding riboprobes (see below). All nucleotide sequences have been deposited in GenBank under the accession numbers MH371150‐MH371155, and ON862499.

### 2.3 BdcRNAs Are Single‐Stranded, Circular, and Highly Paired

The single‐stranded (ss), circular, and highly paired nature of BdcRNAs, similar to viroids, was confirmed by dot blotting hybridization following nuclease digestion with DNase I, S1 nuclease, RNase III, and RNase R and compared with citrus exocortis viroid (CEVd) RNA, extracted from naturally infected citrus seedlings and used as a positive control (Figure S1C, Supporting Information). The enzymatic treatment results for BdcRNAs and CEVd were respectively obtained following dot blotting with antisense riboprobes of BdcRNAs (1, 2.1, and 3.1) and CEVd, labeled with DIG as above respectively (Figure S1D, Supporting Information). Additional controls examined included BdRV1 dsRNAs, remnant genomic DNA, and ribosomal RNAs, co‐extracted from strain XA‐3 (Figure S1C, Supporting Information). The results obtained demonstrated that the RNAs of interest extracted from strain XA‐3, together with CEVd RNAs, were resistant to digestion with DNase I, but partially digested with RNase III, RNase R, and S1 nuclease (Figure S1D, Supporting Information). As anticipated BdRV1 dsRNAs, genomic DNAs, and ribosomal RNAs were digested respectively by RNase III, DNase I, and S1 nuclease and RNase R only (Figure S1C, Supporting Information); the hybridization signals were decreased following RNase R treatment possibly because of the presence of sensitive ssRNA stretches found in the replicative intermediate form of the circular RNAs; S1 nuclease treatment left some trace hybridization signals possibly because of insensitivity of paired nucleotides in some circularised RNAs (Figure S1D, Supporting Information).

### 2.4 BdcRNAs Are Phylogenetically Related but Share No Detectable Homology with Other Known RNAs, and Have Variable Self‐Catalytic Activity

**Figure 2:**
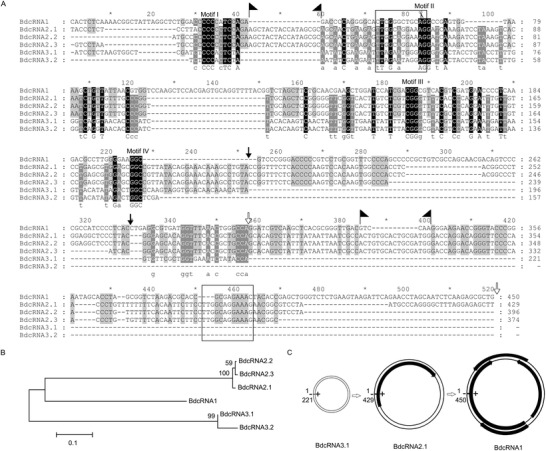
Sequence, phylogenetic and open reading frame (ORF) analysis of BdcRNAs. A) Multiple alignments of BdcRNAs using nucleotide acid sequences from MAFFT version 6.85 as implemented at http://www.ebi.ac.uk/Tools/msa/mafft/ with default settings, except for refinement with 10 iterations. B) Phylogenetic relationships of BdcRNAs. C) Schematic diagram of ORFs harbored by both strands of BdcRNAs. The double circular lines refer to the genome in plus (+) and minus (−), and black blocks refer to potential ORFs with arrows indicating orientation. Black, gray, and light gray backgrounds denote nucleotide identity conserved in all, five and four BdcRNAs, respectively. The arrows and flags denote deletion and insertion delimited positions, respectively, and the scissors denote ribozyme self‐cleavage sites.

BLASTn searches revealed that BdcRNAs 1–3 did not share detectable similarities with any nucleotide sequences deposited in the National Center for Biotechnology Information (NCBI) database or host genomic sequences.^[^ ^11^ ^]^ However significant sequence similarities between the BdcRNAs themselves were noted, for example, 25.6–32.5% (BdcRNAs 1 and 2 or 3), 32.6–68.9% (BdcRNAs 2 and 3), 66.7–97.2% (BdcRNAs 2.1 to 2.3), and 86.1% (BdcRNAs 3.1 and 3.2) (Table S2, Supporting Information). More specifically, BdcRNAs 2.1 to 2.3 differ from each other in deletions of ≈25–30 nt, and BdcRNAs 3.1 and 3.2 in a deletion of ≈100 nt (**Figure** **2**A).

Alignment of the BdcRNAs 1–3 sequences revealed four GC‐rich motifs (I to IV) and a highly conserved 30 nt stretch (indicated with a black background in Figure 2A). Extensive sequence indels were also identified, for example, BdcRNAs 2 contain insertions (_43_AGCUACUACCAUAGCGC_59_ and _386_ACUGUGCACUGCGAUGG_399_, delimited by flags in Figure 2A) absent from the other BdcRNAs. BdcRNA 3 lacks ≈190 nt corresponding to nt 253–329 and nt 357–520 of BdcRNA 2.1 (indicated by arrows in Figure 2A). BdcRNA 2 contains a 12 nt stretch (_73_UUGGCAGGAAAG_85_) directly repeated at nt 452–463, but is absent from BdcRNA 3 while some remnants remain in BdcRNA 1, as indicated by squares in Figure 2A.

**Figure 3:**
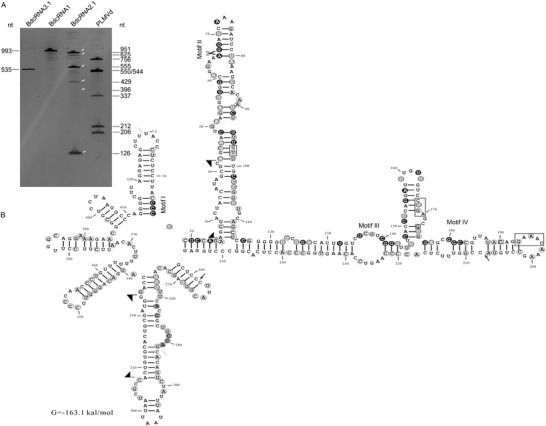
Catalytic activity and secondary structure analysis of BdcRNA 2.1. A) PAGE analysis of in vitro transcription products derived from dimeric cDNAs of BdcRNAs and peach latent mosaic viroid (PLMVd). The numbers refer to the sizes of cleaved products of PLMVd dimeric RNAs or transcribed BdcRNA RNAs, and the arrows indicate cleaved fragments. B) The secondary structures of BdcRNA 2.1 as determined using the RNA structure prediction tool in CLC RNA Workbench software (Version 4.8, CLC bio‐A/S), with conserved motifs and catalytic site indicated in the secondary structure. Black, gray, and light gray backgrounds denote nucleotide identity conserved in all, five and four BdcRNAs, respectively. The arrows and flags denote deletion and insertion delimited positions, respectively, and the scissors denote the self‐cleavage site by the ribozyme.

BdcRNAs 1, 2.1, 2.2, and 2.3 have high G+C contents of 59.3%, 53.9%, 54.3%, and 53.7% respectively. By contrast, BdcRNAs 3.1 and 3.2 have lower G+C contents of 41.6 to 40.1%. Phylogenetic analysis revealed three clusters corresponding to BdcRNAs 1–3 (Figure 2B). The secondary structures of BdcRNAs 1–3, based on the lowest free energy predictions, show simple to complex branched and highly paired conformations differing in the number of loops and branches (**Figure** **3**B and Figure S2, Supporting Information).

Open reading frame (ORF) prediction revealed that BdcRNA 1 contains four potential ORFs, including one in the designated plus (+) strand and three in the minus (−) strand potentially encoding small proteins with estimated molecular masses (Mr) s of 8.7, 3.2, 4.3 and 4.7 kDa, respectively (Figure 2C). A BLASTp search revealed that the largest putative protein has the highest identity (36.6%) with a hypothetical protein of unknown function (AS27_14953) from the emperor penguin *Aptenodytes forsteri* (KFM08303, coverage 51.0%, *e*‐value 2.5). One small ORF was also detected in BdcRNA 2, potentially encoding a protein with an estimated Mr of 6.8 kDa that shows the highest identity (45.7%) with a hypothetical protein of unknown function (Saspl_004291) from the scarlet sage *Salvia splendens* (TEY57791, coverage 53.0%, *e*‐value 4.1). No putative ORFs were detected in BdcRNA 3 (Figure 2C). To check whether the putative proteins encoded by BdcRNAs are bona fide, strains XA3‐MAO‐2‐5 (harboring both BdRV1 and BdcRNA 1), XA3‐MAO‐2‐9 (harboring BdRV1 only) and MAO‐2 were analyzed by LC‐MS after trypsinization. No peptides matching these putative proteins in the above strains were found, while BdRV1 proteins including the RNA dependent polymerase (RdRp) and coat protein were found in both XA3‐MAO‐2‐5 and XA3‐MAO‐2‐9. Overall, these results suggest that the BdcRNAs most likely do not encode any proteins.

The potential self‐cleavage activities of BdcRNAs were examined following electrophoresis on a denaturing PAGE gel and staining with silver nitrate of in vitro transcribed RNA from recombinant pGEM‐T Easy plasmids containing head‐to‐tail dimeric inserts. Dimeric RNAs of peach latent mosaic viroid (PLMVd) containing a HHRz generated by in vitro transcription were used as a control. BdcRNA 2.1 showed self‐cleavage activity resulting in individual RNA products 941, 823, 547, 429, 394, and 118 nt in size, in contrast to both BdcRNA 1 and BdcRNA 3.1 (Figure 3A). RACE, cloning, and sequencing of the smallest cleaved RNA products of BdcRNA 2.1 revealed a catalytic site between nt A_66_ and G_67_ (Figure 3B), within motif II which is conserved in all BdcRNAs (Figure 2A). Sequence analysis suggests that, notably, BdcRNA 2.1 contains a novel ribozyme, which is distinct from all known ribozymes including viroid hammerhead ribozymes.

### 2.5 BdcRNAs Are Horizontally and Vertically Transmitted Independently

Horizontal transmission of BdcRNAs was investigated by contact culture between *B. dothidea* strain XA‐3 as donor and strains MAO‐1 or MAO‐2 as recipients in different combinations (Figure S3AI,II, Supporting Information). Mycelium discs (9‐10) were excised from the colony margins of recipient strains (termed sub‐isolates) and screened for the presence of BdRV1 by dsRNA extraction and electrophoretic fractionation and for the presence of BdcRNAs by northern blotting using antisense riboprobes of BdcRNAs 1, 2.1 and 3.1 as described previously. The results showed that in nine sub‐isolates (MAO‐2‐1 to ‐9), derived from strain MAO‐2 following contact with strain XA‐3, three of the sub‐isolates were infected by two or three BdcRNAs (MAO‐2‐1, ‐2 and ‐5) only. A further four sub‐isolates were infected by two or three BdcRNAs together with BdRV1 (MAO‐2‐3, ‐4, ‐6, and ‐7). Due to incompatibility between strains XA‐3 and MAO‐1 successful combination could only be achieved using strain MAO‐2 as a bridge. Out of ten sub‐isolates obtained when using MAO‐2 as donor and MAO‐1 as recipient (MAO‐1‐1 to ‐10), two sub‐isolates were infected by one to three BdcRNAs (MAO‐1‐5 and ‐7) only, and five sub‐isolates by two or three BdcRNAs together with BdRV1 (MAO‐1‐3, ‐6, ‐8, ‐9 and ‐10) (Figure S3B, Supporting Information). The colony morphology of all sub‐isolates was monitored, and revealed that most displayed phenotypes similar to those of their parental strains as described earlier. However, some sub‐isolates were highly divergent (e.g., MAO‐1‐3 and ‐7, and MAO‐2‐5; Figure S3AIII, Supporting Information) and were not contaminants as adjudged by RT‐PCR amplification of *B. dothidea* ITS sequences and sequencing of the amplicons (results not shown).

To investigate vertical transmission of BdcRNAs, strain XA‐3 conidia were isolated from young fruits following inoculation with mycelium discs. Examination of BdcRNAs by dot blotting (with antisense riboprobes) and the BdRV1 dsRNA profile of 20 randomly selected sub‐isolates of the cultures showed that all were infected by both BdcRNAs 1–3 and BdRV1 (Figure S3C, Supporting Information). These results confirm efficient vertical transmission of BdcRNAs through *B. dothidea* conidia.

### 2.6 BdcRNAs Have Different Subcellular Localizations

**Figure 4:**
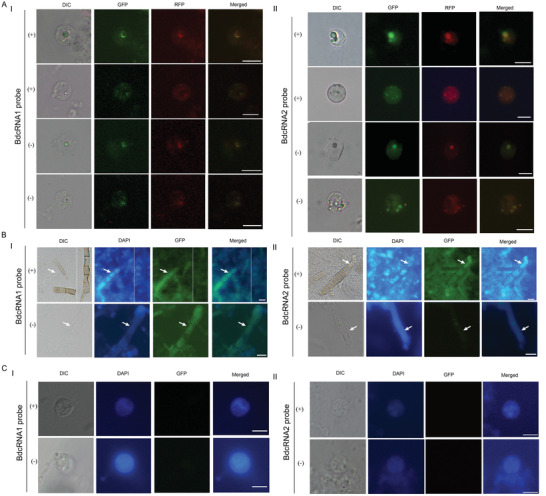
Subcellular location of BdcRNAs. Fluorescence in situ hybridization (FISH) to detect subcellular location of (+) and (−) strands of I) BdcRNA 1 and II) BdcRNA 2.1 A) in protoplasts of strain XA‐3, by Alexa Fluor 488‐labeled riboprobes (green fluorescence), B) as well as in newly formed mycelia containing no assembled nuclei, with strain MAO‐2 protoplasts investigated in parallel as controls C). Protoplasts containing one and more nuclei are shown under differential interference contrast (DIC) or fluorescent settings. Nuclear location was indicated with mCherry, binding to the fungal nucleosome (red fluorescence), or stained with DAPI blue color at a higher concentration than in the cytoplasm. The fungal cells contain no nuclei or one or more nuclei depending on age and those nuclei in the process of assembling were not extensively labeled with mCherry or stained with DAPI. All microscope images were photographed at 400× magnification. Scale bars = 10 µm.

To gain insight as to the subcellular locations of the BdcRNAs, fluorescence in situ hybridization (FISH) was performed with strain XA‐3 protoplasts using Alexa Fluor 488‐labeled riboprobes specific for (+) and (‐) strands of BdcRNA1 and BdcRNA2.1. Both strands of BdcRNA1 were present predominantly in regions peripheral to nuclei (**Figure** **4**AI), whereas these of BdcRNA 2 were concentrated inside nuclei (Figure 4AII), as shown by green fluorescence produced by the riboprobes overlapping red fluorescence produced by mCherry, which binds to fungal histones, and blue coloration following staining with diamidine phenylindole (DAPI; Figure 4A). Conversely, in some cells, both strands were absent from nuclei but still diffused into the cytoplasm (Figure S4A,B, Supporting Information). In mycelia, both strands of BdcRNA 1 and the (+) strands of BdcRNA 2 but not the (−) strands spread systemically and uniformly in growing or newly formed mycelia where nuclei were yet to mature (Figure 4B). No hybridization signals were found in strain MAO‐2 cells which do not harbor BdcRNAs (Figure 4C).

### 2.7 BdcRNAs Replicate via a Rolling‐Circle Mechanism Following Symmetric Pathways In Vivo

**Figure 5:**
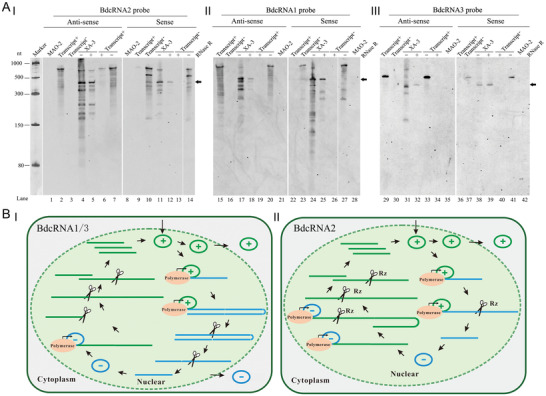
Replication analysis of BdcRNAs. A) Northern blotting hybridization analysis of nucleic acid preparations from strains MAO‐2 and XA‐3 using antisense and sense riboprobes of BdcRNAs I) 2.1, II) 1, and III) 3.1, respectively. Marker 1, an ssRNA marker electrophoresed on the same gel was visualized by staining with silver nitrate. The (+) and (−) ssRNAs of BdcRNAs derived by in vitro transcription were examined were used to assess probe specificity and confirm lack of cross hybridization between strands (lanes 2, 3, 9, 10, 15, 16, 22, 23, 29, 30, 36, and 37). The nucleic acid preparations from strain XA‐3 were also subjected to digestion with RNase R to confirm the circular nature of the RNAs, together with the transcripts binding to the riboprobes as controls. In this case “+” and “‐” refer to the presence and absence of RNase R, respectively. Lanes are numbered below the panels. The arrows indicate circular forms. B) Proposed symmetric models of a rolling‐replication pathway for I) BdcRNAs 1 and 3, and II) 2 in a single cell, respectively. Green and blue lines (linear forms) or cycles (circular forms) indicate (+) and (‐) strands, respectively, and their sizes correspond to the RNA polymeric units. The strands are synthesized by a hypothetical polymerase (indicated by orange oval blocks), cleaved by unknown host factors (indicated by scissors), or by a ribozyme (Rz), and the artificial replication process is indicated by arrows. The symmetric pathway, with two rolling circles, takes place in the nucleus, and is delimited from the cytoplasm by green dot lines.

DIG‐labeled, full‐length, sense, and antisense BdcRNAs 1, 2.1, and 3.1 riboprobes were prepared and their specificity was examined by northern blotting with both sets of transcripts loaded at the same concentration (**Figure** **5**A). The results showed that all DIG‐labeled riboprobes bind with antisense transcripts (Figure 5A, lanes 2, 10, 15, 23, 29, and 37), and did not bind sense transcripts (lanes 3, 9, 16, 22, 30, and 36) or nucleic acids from strain MAO‐2 (lanes 1, 8, 21, 28, 35, and 42), These results illustrate that all of these riboprobes were high specific under the hybridization conditions used. Total RNA preparations extracted from strain XA‐3 were treated with or without RNase R, electrophoresed on a denaturing PAGE gel, electro‐transferred to a nylon membrane, and subjected to blot analysis using similar riboprobe amounts. An ssRNA marker co‐electrophoresed with the RNA preparations separately was stained with silver nitrate to estimate RNA fragment sizes. The results revealed that BdcRNAs 1–3 (+) strands accumulate abundantly in vivo in circularised forms, which are resistant to RNase R digestion and correspond to the 450, 429, and 221 nt fragments displayed on gels following fractionation by PAGE (Figure 5A, lanes 5, 18, and 32). Dimeric (BdcRNAs 1 and 2; lanes 4 and 17 respectively) or trimeric forms (BdcRNA 3; lane 31) were also present but at significantly lower titers as compared to the circular forms following reference to the ssRNA marker (Figure 5AI, panel left). Antisense BdcRNAs 1–3 also accumulated in circular forms (Figure 5A, lanes 12, 25, and 39) together with dimeric forms (BdcRNAs 1 and 2; lanes 11 and 24 respectively) or oligomeric forms (BdcRNA3; lane 38). Taken together the analyses of the subcellular localization and polarity of the circular RNAs described above suggest that BdcRNA replication in vivo is symmetric and similar to that adopted by chloroplastic viroids (Figure 5B): In detail, a circular (+) strand is transcribed to yield a dimeric (for BdcRNAs 1 and 2) or an oligomeric (for BdcRNA 3) linear (−) strand, which is cleaved by host protein(s) (for BdcRNAs 1 and 3; Figure 5BI) or by a ribozyme (for BdcRNA 2; Figure 5BII) to unit lengths and then circularized in the nucleus. The resulting (−) circular strand serves as a template to produce (+) dimeric (for BdcRNAs 1 and 2) or oligomeric forms (for BdcRNA 3), which are then cleaved and circularized in the nucleus. The resulting circular (+) strands are then transported into the cytoplasm (Figure 5B).

### 2.8 Artificial BdcRNAs Replicate Autonomously and Spread Systemically In Vivo

To assess the infectivity and replication of individual BdcRNAs, in vitro transcripts derived from dimeric head‐to‐tail cDNAs of BdcRNA 1, 2.1, and 3.1 were transfected into MAO‐2 protoplasts resulting in respectively 13, 4, and 11 positively infected sub‐isolates as assessed by dot blotting and RT‐PCR amplification (Figure S5, Supporting Information). Three transfectants (T11, T32, and T14 transfected with BdcRNAs 1, 2.1, and 3.1, respectively) were selected for protoplast isolation, and twenty single cells, designated T11‐1 to ‐20 (for BdcRNA 1), T32‐1 to ‐20 (for BdcRNA 2.1), and T14‐1 to ‐20 (for BdcRNA 3.1), were serially passaged over six generations. The BdcRNA titers of the transfectants, together with their parent strains (T11, T32, and T14), were assessed by RT‐qPCR amplification (flowchart, Figure S6A, Supporting Information). Based on the quantitative absolute standard curves established with serially diluted plasmids containing BdcRNA cDNAs (Figure S6B, Supporting Information), RT‐qPCR amplification revealed that BdcRNA1 titers fluctuated (1.2‐1.7 × 10^4^ copies µL^−1^) in the progeny (T11‐1 to ‐20) without any significant difference as compared to the titer (1.5 × 10^4^ copies µL^−1^) of the parent isolate (T11) (Figure S6CI, Supporting Information). Results for BdcRNA 2.1 and BdcRNA 3.1 (T32‐1 to‐20 and T14‐1 to ‐20) were similar, but fluctuations were much more pronounced, and progeny with substantially higher or lower titers were recorded (Figure S6CII,III, Supporting Information). These results suggest that BdcRNAs can replicate autonomously and spread systemically into new, growing cells, but that their accumulated titers differ between individual cell generated colonies.

### 2.9 BdcRNAs Accumulation Is Not Affected by Co‐Infection with Mycoviruses

To determine whether replication of BdcRNAs is affected by BdRV1 co‐infection, BdRV1 was introduced into sub‐isolate T11 using horizontal transmission. Three of 10 sub‐isolates examined were successfully infected with BdRV1 (BdRV1‐T11‐C5 to ‐C7) as assessed by dsRNA extraction and gel electrophoresis (Figure S7AI, Supporting Information). Examination of the titers of BdcRNA 1 in the three sub‐isolates above, as quantified by RT‐qPCR amplification, varied but were not significantly different from the parental sub‐isolates (Figure S7AII, Supporting Information).

In order to determine if infection of *B. dothidea* containing BdcRNAs with a different mycovirus altered RNA titer similar experiments to those described above were performed. Here, Botryosphaeria dothidea partitivirus 1 (BdPV1) was successfully transfected into protoplasts of sub‐isolates T11 (BdcRNA 1^+^) and T32 (BdcRNA 2.1^+^) as demonstrated by dsRNA isolation and electrophoresis (Figure S7B, Supporting Information). Ten transfectants from each sub‐isolate were assessed by RT‐qPCR amplification revealing titers in the BdPV1‐infected sub‐isolates similar to those of the parental sub‐isolates (Figure S7CI,II, Supporting Information). In conclusion these results suggest that mycovirus co‐infection had no obvious effects on the accumulation of BdRNAs in vivo.

### 2.10 Individual BdcRNA(s) Modulate Specific Biological Traits of the Host Fungus

**Figure 6:**
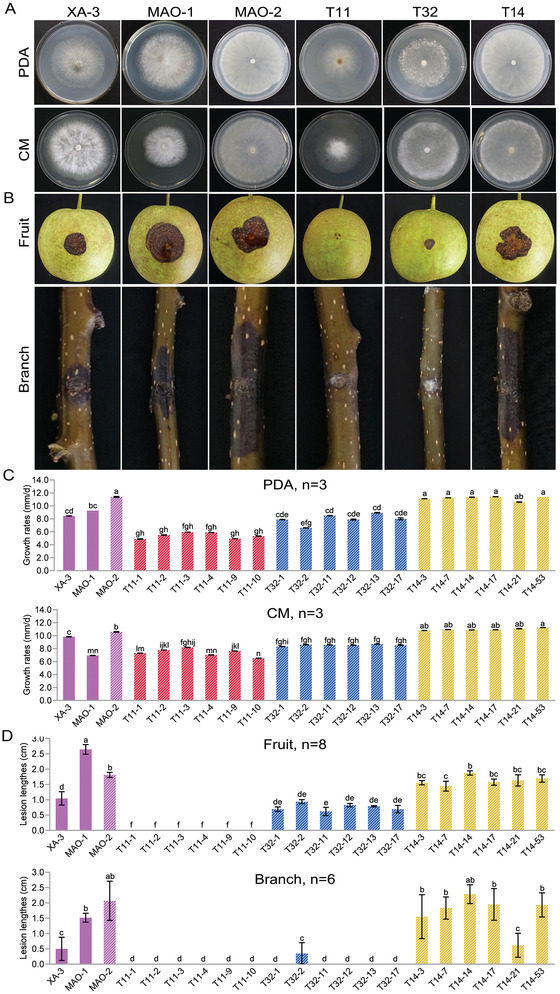
Morphology, growth rate, and virulence of transfectants of *B. dothidea* MAO‐2 infected with BdcRNAs together with control strains. A) Representative morphology of the transfectants derived from BdcRNAs 1 (T11‐), 2.1 (T32‐), and 3.1 (T14‐) on PDA and complete medium (CM). B) Virulence assessment on pear (var. Cuiguan) fruits (*n* = 8) and tree branches (*n* = 6) for 7 days, respectively. C,D) Histograms for the growth rates assessment on PDA, and the virulence assessment on pear fruits, respectively. Bars in each histogram labeled with the same letters are not significantly different (*P* > 0.05) according to the least significant difference test (1‐way ANOVA); and error bars represent SD.

To better understand the biological roles of individual BdcRNAs, six BdcRNA 1, 2.1, and 3.1 transfectants, together with XA‐3, MAO‐1, and MAO‐2, were grown on PDA and complete medium (CM). Examination of BdcRNA 1 and BdcRNA 2.1 transfectants (T11‐1 to ‐10, and T32‐1 to ‐17) revealed altered morphology as compared to the parental strain MAO‐2 where the former produced dense mycelia, while the latter produced sparse mycelia with irregular margins. In contrast, BdcRNA 3.1 transfectants (T14‐3 to ‐53) showed no obvious phenotypical changes (**Figure** **6**A). Moreover, the growth rates of both BdcRNA 1 and BdcRNA 2.1 transfectants were significantly decreased whilst the growth rate of BdcRNA 3.1 transfectants was unaffected (Figure 6C). BdcRNA 1 virtually eliminated fungal virulence as assessed on pear fruits and branches, since no lesions were observed following transfectant infection while inoculation with the parent MAO‐2 strain resulted in lesions ≈1.8 cm in size on fruits and ≈2.1 cm on branches. BdcRNA 2.1 transfectants also exhibited significantly decreased virulence on fruits and no virulence for branches apart from sub‐isolate T32‐2 whereas BdcRNA 3.1 transfectants did not demonstrate attenuated virulence on fruits and branches apart from sub‐isolates T14‐7 and ‐21 (Figure 6B,D). These results suggest that BdcRNAs play diverse but well‐defined roles in the modulation of host morphology, growth, and virulence.

**Figure 7:**
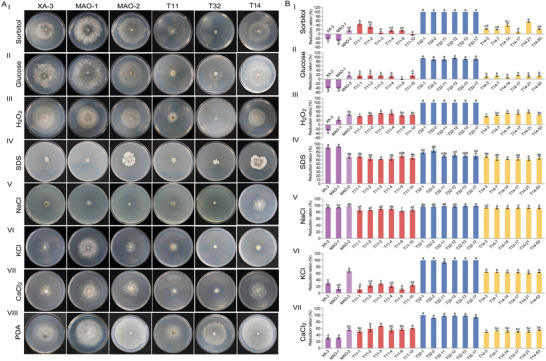
Stress assessment of BdcRNAs 1 (T11‐), 2.1 (T32‐), and 3.1 (T14‐) transfectants from *B. dothidea* strain MAO‐2 on various media together with control strains. A) Growth assessment on PDA amended with I) 1 mol L^−1^ sorbitol, II) 1 mol L^−1^ glucose, III) 10 mm H_2_O_2_, IV) 0.005% SDS, V) 1.5 mol L^−1^ NaCl, VI) 1 mol L^−1^ KCl, VII) 0.5 mol L^−1^ CaCl_2_, and VIII) PDA incubated at 25 °C in darkness for 3 days, respectively. B) Histograms of growth reduction rates on amended media (I to VII) as compared to growth on PDA (*n* = 3). Bars on each histogram labeled with the same letters are not significantly different (*P* > 0.05) according to the least significant difference test (1‐way ANOVA) Error bars represent SD.

To determine the role of BdcRNAs in fungal stress responses, transfectants were cultured on PDA amended with various cellular stress agents, including those involved in sugar metabolism (1 mol L^−1^ sorbitol and 1 mol L^−1^ glucose), cell wall disruption (0.005% SDS), oxidative stress (10 mm H_2_O_2_), osmotic stress (1 mol L^−1^ KCl and 1.5 mol L^−1^ NaCl), and the calcium pathway (0.5 mol L^−1^ CaCl_2_). All BdcRNA 1 transfectants (T11‐1 to ‐10) showed significantly higher tolerance to high concentrations of KCl and NaCl as compared to the parent strain MAO‐2. BdcRNA 2 transfectants showed strong sensitivity to high concentrations of sorbitol, glucose, H_2_O_2_, KCl, and CaCl_2_ and BdcRNA 3 transfectants (T14‐3 to ‐53) only showed tolerance to high concentrations of NaCl (**Figure** **7**A,B). These results showed that individual BdcRNAs interfere with *B. dothidea* and exert diverse regulatory roles in vivo under different stress conditions.

### 2.11 BdcRNA(s) Modulate Gene Expression and Metabolism of the Host Fungus

**Figure 8:**
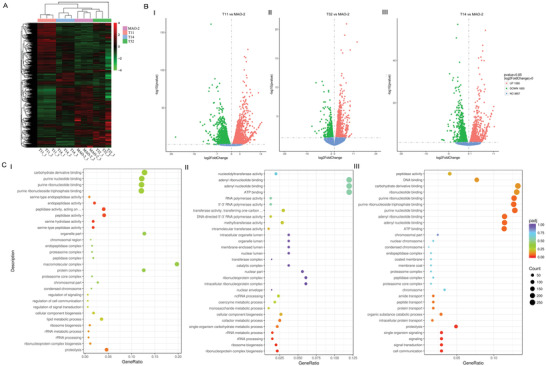
Transcriptome analyses of transfectants of *B. dothidea* MAO‐2 infected with BdcRNAs together with the control strain. A) Clustered heat map of differentially expressed genes (DEG) of transfectants infected by BdcRNAs 1 (T11), 2.1 (T32), and 3.1 (T14) together with MAO‐2. B) Volcano plots of DEG of transfectants T11, T32, and T14 as compared with MAO‐2. C) Scatter diagrams of GO enrichment analysis of DEG of transfectants I) T11, II) T32, and III) T14.

To analyze the effect of BdcRNAs on host gene expression, three transfectants together with the parental strain of each BdcRNA were grown on PDA for 8 days and then subjected to transcriptome profiling by next‐generation sequencing (NGS) and differential gene expression analysis. The results revealed distinct gene expression patterns modulated by BdcRNAs 1–3 as compared to the control strain MAO‐2 according to heat map analysis (**Figure** **8**A), with 6741, 2122, and 3400 differentially expressed genes (DEGs) (Figure 8B), accounting respectively for 47.8%, 15% and 24.1% of the total genes of the host fungus (14 116 genes in the genome, ID: 12428). Gene ontology (GO) classification revealed the DEGs were mainly involved with peptidase metabolic pathways, including peptidase, endopeptidase, serine‐type endopeptidase, and serine‐type peptidase activities for BdcRNA 1 transfectants (Figure 8CI), ribosome metabolic pathways (rRNA metabolic process, rRNA processing, ribosome biogenesis, and ribonucleoprotein complex biogenesis) for BdcRNA 2 transfectants (Figure 8CII), and proteolysis, signaling and cell communication for BdcRNA 3 transfectants (Figure 8CIII).

To verify the transcriptome results, six downregulated DEGs, including these of the glycosyl hydrolase family (ID A08082), the glycosyl transferase family (ID A03050), and some of unknown functions (A07378, A06265, A03482, and A09837), were examined by RT‐qPCR in MAO‐2 transfectants (T11 and T32) at 6, 8, and 10 days post inoculation on PDA. All selected genes showed decreased expression patterns similar to those obtained by transcriptome profiling at no less than one time point, supporting the findings of the NGS results (Figure S8, Supporting Information).

## 3 Discussion

In this study, we have identified a set of circular ssRNAs, BdcRNAs 1–3, in *B. dothidea* strain XA‐3. Understanding the nature of these molecules was based on the following evidence: i) 2D‐PAGE in combination with northern blotting analysis revealed that BdcRNAs migrated slower than host RNAs of comparable size, ii) BdcRNAs were resistant to DNase I, RNase R, and RNase III, and iii) RT‐PCR amplification using abutted primer pairs amplified full‐length sequences. BdcRNAs were capable of horizontal transmission, independent of each other or mycoviruses, and autonomous replication following transfection.

BdcRNAs display a rapid genomic expansion including insertions, mutations, and deletions, and they show remarkable variation in nucleic acid sequence, size (157–450 nt), secondary structure complexity, GC content, and potential protein coding capacity. However, BdcRNAs share nucleotide identities including four conserved GC‐rich motifs and a highly conserved stretch >30 nt in length (Figure 2A). These observations support evolution of BdcRNAs from a common ancestor. Notably, BdcRNAs 1 and 2 contain potential ORFs, which clearly separate them from BdcRNA 3 (and viroids); however, no resulting proteins were detected by proteomics analysis, which might provide a link between noncoding and coding RNAs. Since the properties of BdcRNAs are those expected for primitive RNA replicons, including their small size (BdcRNA 3.1), high GC content (up to 59.3% for BdcRNA 1), circular structure, structural periodicity, lack of coding capacity (for BdcRNA3), and a novel ribozyme (for BdcRNA 2), that is, the fingerprints of the RNA world,^[^ ^1a^ ^]^ BdcRNAs may represent a link between primordial RNAs and modern coding RNAs.

BdcRNAs share no detectable sequence identities with any of these endogenous cRNAs, or with the host genome, and are not detectable in virulent *B. dothidea* strains, suggesting that they are exogenous cRNAs. The known exogenous small cRNAs in eukaryotic cells include small satellite RNAs and viroids. Satellite RNAs have genomes ≈350 nt in length, infect plants, and are associated with plant viruses. Two small circular RNAs (cscRNA 1 and cscRNA 2) isolated from cherry trees were found in combination with mycoviral dsRNAs and it has been suggested that they are viroid‐like satellite RNAs.^[^ ^12^ ^]^ BdcRNAs did not co‐sediment with BdRV1 following sucrose gradient (10% to 40%) centrifugation and no additional mycoviral genomes were detected either (data not shown). These observations suggest that the circular RNAs are independently infectious, and their replication is unaffected by mycoviruses. Moreover, the genomes of most satellite RNAs have short sequences identical to its helper virus genome. Also, their termini possess unique, conserved sequences which play an important role in viral packaging, transcription, and replication.^[^ ^13^ ^]^ It is considered unlikely that mycoviruses host the BdcRNAs which are variable in size and sequence similarity. These results suggest that BdcRNAs are not satellite RNAs or viroid‐like satellite RNAs but another type of infectious, autonomous replicating agent resembling viroids. The BdcRNAs resemble plant viroids in terms of size, circularity, and infectivity, but they lack the CCR and HHRzs typical of nuclear and chloroplastic viroids, respectively. Additionally, BdcRNAs have strands located in both the nucleus and the cytoplasm with distinct distribution patterns, significantly different from the subcellular location of *Pospiviroidae* and *Avsunviroidae* viroids, which are located in the nucleus and the chloroplast, respectively, with both of their strands (as demonstrated in CEVd, coconut cadang–cadang viroid, and PSTVd) exhibiting similar distribution patterns.^[^ ^14^ ^]^ Furthermore, although BdcRNAs 1 and 3 contain complex branched secondary structures similar to those of chloroplastic viroids, they possess no ribozyme and are similar to nuclear viroids.^[^ ^1^, ^15^ ^]^ BdcRNA 2 contains a novel ribozyme, which is distinct from other ribozymes including the HHRzs. Therefore, BdcRNAs may represent a novel class of autonomously replicating subviral agents which are not satellites but resemble plant viroids. In previous studies, avocado sunblotch viroid (ASBVd) was shown capable of infecting *Saccharomyces cerevisiae* with dimeric/oligomeric cDNAs fused to an expression vector.^[^ ^3a^ ^]^ Also ASBVd, hop stunt viroid (HSVd), and iresine viroid‐1 (IrVd‐1) were each successfully transfected with monomeric transcripts into three filamentous phytopathogenic fungi (*Cryphonectria parasitica*, *Valsa mali*, and *Fusarium graminearum*). These results support the notion that viroids could replicate in at least one of these three fungi,^[^ ^3^, ^16^ ^]^ although this study has stimulated some controversy since the major evidence for replication in the fungi was obtained by RT‐PCR amplification. These results need to be verified using additional detection methods and appropriate controls.^[^ ^3^, ^17^ ^]^ No natural infections of fungi with viroids or viroid‐like RNAs have been reported in previous studies^[^ ^18^ ^]^ and, to our knowledge, this is the first report of infectious, viroid‐like RNAs (or exogenous small circular RNAs) in a life kingdom (fungi) other than plants. Here, the term “mycoviroid” was tentatively used for such types of novel acellular entities with reference to viroid‐like RNAs naturally infecting fungi, expanding the term beyond its original definition as “a viroid that has the ability to infect healthy fungi.”^[^ ^19^ ^]^

FISH experiments revealed that the BdcRNAs have different distribution patterns in both cells and mycelia depending on the species under investigation and RNA polarity. High concentrations of both strands in the nucleus may reflect their localization during replication and transcription. The absence of both strands of BdcRNAs 1 and 2 from nuclei observed in some cells is likely caused by a cessation of replication due to cell aging and the export of synthesized cRNAs out of the nuclei. Correspondingly, BdcRNA 2 (+) strands instead of (−) strands were translocated from older to younger hyphae. Detection of the (+) and (−) dimeric or oligomeric forms together with circular forms of both polarities suggests that BdcRNAs 1–3 follow a symmetric rolling‐circle replication mechanism in vivo (Figure 5B), similar to previous reports for viroids.^[^ ^20^ ^]^ The absence of oligomeric forms of BdcRNAs 1 and 2 might be due to rapid self‐cleavage during elongation which is dissimilar to ASBVd which replicates using a symmetric replication pathway^[^ ^21^ ^]^ Currently there is no information concerning the mechanisms of replication and movement of exogenous cRNA in fungi. Here we demonstrate for the first time that exogenous small cRNAs replicate, are transported, and distributed in fungal cells in diverse ways depending on the species and strand polarity. These observations support the notion that fungi have developed machinery which can recognize and localize diverse types of small cRNAs in a strand specific manner.

BdcRNAs significantly affect the biological traits of *B. dothidea*, for example, alter morphology, decrease growth rate, attenuate virulence, and increase or decrease tolerance to osmotic stress and oxidative stress. Concurrent with phenotype modulation, gene expression, and metabolic pathways related to important cellular processes of the fungal host were also modulated by BdcRNAs. For BdcRNA 1 and 2 transfectants, all related RNA processing genes were significantly down regulated, and these changes may provide an explanation as to why BdcRNA‐infected strains attenuate growth rates. RNA processing is important in biology, involving nucleic acid metabolism, gene expression, protein metabolism, and cellular component biogenesis,^[^ ^22^ ^]^ all of which play crucial roles in fungi. Moreover, the DEGs related to peptidase activities were significantly down regulated for BdcRNA 1 transfectants. These observations may explain their attenuated virulence since peptidases (proteases) contribute to both fungal growth and pathogenesis, and are considered markers of pathogenicity.^[^ ^23^ ^]^ Virulence was differentially attenuated in all BdcRNA transfectants, most likely due to the extent of differential expression of related genes. Thus far, mycoviruses are the only acellular agents that have been extensively investigated and infect all major fungal taxa. Here we describe another acellular entity, distinct from mycoviruses, which confers important effects on the physiology and pathogenicity of the host fungus, as exemplified by BdcRNA 1 which dramatically reduces host virulence. This feature provides an important alternative candidate to serve as a biocontrol tool for the attenuation of fungal diseases similar to some mycoviruses that cause hypovirulence.^[^ ^24^ ^]^

In summary, this work shows that a set of BdcRNAs display molecular and biological features unreported in known living entities, and may represent a new class of viroid‐like RNAs endowed with regulatory functions, and novel epigenomic carriers of biological information. BdcRNAs contribute to the diversity of fungal biological traits, and may help us understand fungal diversity and biological changes along the life process besides genomics of fungi or even other cellular organisms. Moreover, BdcRNAs can be regarded as additional inhabitants of the frontier of life in terms of genomic complexity, and may represent another group of “living fossils” from a precellular RNA world as compared with viroids.^[^ ^1^, ^25^ ^]^ More importantly, BdcRNAs can attenuate the virulence of phytopathogenic fungi, and may be exploited as biocontrol agents. Here, “mycoviroid” was termed for such viroid‐like RNAs naturally infecting fungi besides its original definition,^[^ ^19^ ^]^ with BdcRNAs 1 and 2 as the type species of two separate groups.

## 4 Experimental Section

### Fungal Isolates and Biological Characterization

*B. dothidea* strains XA‐3, MAO‐1 and MAO‐2 were isolated from apple tree branches (*Malus domestica* Borkh. cv. “Fuji”) collected in Shandong province, China and were identified based on morphological characteristics and molecular analyses. All strains were purified by hyphal‐tipping.^[^ ^26^ ^]^

### RNA Extraction and Enzymatic Treatments

For dsRNA extraction, mycelial plugs were inoculated onto cellophane membranes on potato dextrose agar (PDA; 20% diced potatoes, 2% glucose, and 1.5% agar) plates and incubated in darkness at 25 °C for 4 to 5 days. ≈1 g mycelia were collected, ground to a fine powder in liquid nitrogen, and subjected to dsRNA extraction as previously described.^[^ ^10^ ^]^Briefly, the frozen powder was treated with five volumes of buffer (2% SDS, 4% PVP‐40, 0.5 m NaCl, 100 mm Tris‐HCl ([pH 8.0], 20 mm EDTA), and an equal volume of binding buffer (50% guanidine thiocyanate, 1.5 m KCl, 0.5 m NH_4_Cl, 0.3 m KAcO [pH 6.0]. The resulting mixture was centrifuged at 12 000 × *g* for 5 min. The supernatant was mixed with 0.5 volumes of ethanol, and loaded onto a silica spin column (Sangon Biotech (Shanghai) Co., Ltd, China) to absorb the dsRNA, which was eluted with 60 µL RNase‐free water after two washes with the binding buffer containing 37% ethanol.

Alternatively, RNAs with compact secondary structure were extracted as previously described.^[^ ^8^ ^]^ Briefly, 30 g of frozen powdered mycelia was treated with buffer‐saturated phenol (pH 7.0), and following centrifugation at 12 000 × *g* the supernatant was mixed with non‐ionic cellulose (CF‐11, Whatman) in 1× STE buffer (50 mm Tris‐HCl (pH 7.2), 100 mm NaCl and 1 mm EDTA) and 30% ethanol and shaken overnight. After washing the cellulose three times with STE containing 35% ethanol, bound RNAs were eluted with 1× STE, precipitated with ethanol, dissolved in 60 µL RNase‐free water, and stored at −70 °C until use.

The RNA preparation from strain XA‐3 together with circular and linear CEVd RNAs, extracted from a citrus tree infected with a CEVd variant CEVd.188,^[^ ^27^ ^]^ were subsequently treated with several enzymes to determine their nucleic acid nature. Briefly, aliquots of 200 ng nucleic acid were treated with 2 U DNase I (Thermo Scientific), 10 U S1 nuclease (Thermo Scientific), or 0.4 U RNase III (New England Biolabs) in cleavage buffer [30 mm Tris‐HCl (pH 8.0), 160 mm NaCl, 0.1 mm EDTA, 0.1 mm DTT, 10 mm MgCl_2_], as previously described,^[^ ^10^ ^]^ and with 200 ng mL^−1^ RNase R (Geneseed) in 2× SSC (300 mm NaCl, 30 mm sodium citrate, pH 7.0) at 37 °C for 1 h according to the manufacturer's instructions. After enzyme treatment nucleic acids were treated with phenol/chloroform/isoamyl alcohol (25:24:1) (phenol saturated with water, pH 5.2), nucleic acids precipitated with ethanol, dissolved in RNase‐free water, and stored at −70 °C until use.

The nucleic acid preparations were analyzed by 6% non‐denaturing PAGE or 1.2% agarose gel electrophoresis and visualized by staining with silver nitrate and ethidium bromide, respectively.

### cDNA Synthesis and Molecular Cloning

The cDNA sequences of the five dsRNAs identified on gels were determined as previously described.^[^ ^28^ ^]^ The 5′ and 3′ terminal sequences of the dsRNAs were determined using RNA ligase mediated rapid amplification of cDNA ends (RLM) RACE.^[^ ^28^ ^]^

Circular RNA cDNA sequences were determined using a random cloning method as previously reported.^[^ ^10^ ^]^ Briefly, purified cRNAs were subjected to cDNA synthesis using M‐MLV reverse transcriptase with Primer I (5′‐GAC GTC CAG ATC GCG ATT TCN NNN NN‐3′), and amplified using Primer II (5′‐GAC GTC CAG ATC GCG ATT TC‐3′) in combination with end filling with *Taq* polymerase. The amplified PCR products were cloned into the pMD18‐T vector (TaKaRa, Dalian, China) and transformed into *Escherichia coli* DH5alfa competent cells. At least three independent clones of each fragment were sequenced in both directions. Sequencing was performed at Sangon Biotech (Shanghai) Co., Ltd, China, and each nucleotide was determined in at least three independent overlapping clones in both orientations.

Further RT‐PCR identification of circular RNAs was conducted using primers designed based on assembled contigs (Table S1, Supporting Information) of cDNAs synthesized using random hexamers as described above. Amplification was performed in a thermal cycler for 35 cycles of 94 °C for 30 s, 50–60 °C for 30 s, and 72 °C for 30 s after initial denaturation at 95 °C for 30 s, and followed by a final extension at 72 °C for 10 min.

### Sequence Analysis and Self‐Cleavage Activity

Sequence similarity searches were performed using the NCBI databases with the BLAST program. Multiple alignments of nucleotide and amino acid sequences were conducted using MAFFT version 6.85 as implemented at http://www.ebi.ac.uk/Tools/msa/mafft/ with the default settings except for refinement with 10 iterations. Identity analyses were conducted using the DNAMAN DNA analysis software package (DNAMAN version 6.0; Lynnon Biosoft, Montreal, Canada). The secondary structures of BdcRNAs were determined using the RNA structure prediction tool in CLC RNA Workbench software (Version 4.8, CLC bio A/S) with the algorithms as described.^[^ ^29^ ^]^ ORFs were deduced using ORF Finder online (https://www.ncbi.nlm.nih.gov/orffinder/).

The PCR products generated from the full‐length of BdcRNAs were dimerized and ligated into the pGEM‐T Easy vector as described previously.^[^ ^30^ ^]^ The constructed plasmids were sequenced to confirm that they harbored two head‐to‐tail copies of the original version of BdcRNA cDNAs, digested with *Nde* I (TaKaRa Biotechnology Corp., Dalian, China), and used for in vitro transcription by T7 RNA polymerase (Thermo Fisher Scientific Inc., Shanghai, China) as described previously.^[^ ^30^ ^]^ Transcripts were separated in a 6% polyacrylamide gel containing 8 m urea and stained with silver nitrate solution to access their self‐catalytic activities. To access self‐catalytic sites, the in vitro transcription products were fractionated in a denaturing polyacrylamide gel and purified as previously described,^[^ ^27^ ^]^ dissolved in water, and the 5′‐terminal sequences of the 3′‐cleavage products determined by RLM RACE.

### 2D‐PAGE, Probe Preparation, and Hybridization

2D‐PAGE was conducted as previously described,^[^ ^8^, ^9^ ^]^ with some modifications. Briefly, aliquots were first separated by non‐denaturing PAGE in a 5% gel in 1× TAE (40 mm Tris‐HC1, 20 mm NaAcO, 2 mm EDTA, pH 7.5) and stained with ethidium bromide. A gel fragment, delimited by DNA markers 100 and 500 bp in size, was excised and placed on top of a second denaturing 5% gel in 0.25× TBE (where 1× TBE buffer was 89 mm Tris, 89 mm H_3_BO_3_, 20 mm EDTA, pH 8.0) and 8 m urea. Following electrophoresis circular RNAs migrated slowly as compared to their linear counterparts.^[^ ^8^, ^9^ ^]^

DIG‐labeled full‐length riboprobes complementary (antisense) or homologous (sense) to BdcRNAs 1, 2.1, and 3.1 were prepared as previously described.^[^ ^8^, ^31^ ^]^ Nucleic acids were spotted (for dot blotting) or electro transferred (for northern blotting) to positively‐charged nylon membranes (Roche Diagnostics), and hybridized with DIG‐labeled full‐length riboprobes as previously described.^[^ ^8^, ^31^ ^]^

The potential self‐catalytic activities of BdcRNAs were examined using RNA transcribed from recombinant plasmids containing dimeric inserts.

### Fluorescence In Situ Hybridization

FISH was conducted essentially as described before,^[^ ^14^ ^]^ using riboprobes labeled with Alexa Fluor 488 following the approach described below for DIG‐labeled riboprobes. All samples were stained with the DNA fluorochrome 4′,6‐diamidino‐2‐phenylindole dihydrochloride (DAPI) dissolved in PBS at a final concentration of 1 µg mL^−1^ for 5 min. Additionally, strains MAO‐2 and XA‐3 were transfected with the pBS‐NEO‐Histone‐mCherry plasmid to identify fungal histones in the nucleus. The excitation and emission wavelengths were respectively 330 to 380 nm for DAPI, 450 to 490 nm for Alexa Fluor 488, and 587 to 610 nm for mCherry.

### Preparation of Protoplasts and Transfection of BdcRNAs

Protoplasts were prepared from actively growing mycelia of the corresponding strains as previously described^[^ ^32^ ^]^ and aliquots were stored at −70 °C until use.

Protoplasts were filtered through a Millipore filter, counted using a hemocytometer, and used for BdcRNA transfection using PEG 6000 as previously described.^[^ ^33^ ^]^ A total of 1.0 × 10^6^ protoplasts were transfected with ≈5.0 µg of in vitro transcribed dimeric BdcRNAs RNAs. Following transfection protoplast suspensions were diluted with sterilized water and spread onto PDA plates and any new colonies were separately cultured on fresh PDA plates for BdcRNA extraction.

### Morphology, Growth Rate, Stress‐Resistance, and Virulence Assays

Morphology and growth rate were estimated in triplicate at 25 °C following incubation in darkness for 3 days as previously described.^[^ ^10^ ^]^ The stress‐resistance assay to salt and osmotic pressure was conducted by assessment of the growth rates at 25 °C in the dark on PDA by incorporating the corresponding components at a suitable concentration to the medium after assessment of serial dilutions. The virulence of each strain was determined by inoculating detached fruits (eight replicates) or branches (six replicates) of *M. domestica* cv.“Fuji” or *Pyrus pyrifolia* cv. “Cuiguan” as previously described.^[^ ^34^ ^]^

### Quantitative Real‐Time PCR

Quantitative real‐time PCR (RT‐qPCR) to measure BdcRNA titer was conducted using the CFX96 Real‐Time PCR Detection System (Bio‐Rad, USA) based on the cDNAs synthesized as described above using specific primers designed for quantitative analysis (Table S1, Supporting Information). Amplification was conducted in a thermal circular for 45 cycles of 95 °C for 30 s, 60 °C for 20 s and 72 °C for 20 s after initial heating at 95 °C for 3 min, and followed by a final extension for 10 min at 72 °C. A fragment of the *B. dothidea* actin gene was used to normalize the RNA samples for each RT‐qPCR, and each treatment was conducted with three technical replicates. The absolute quantitative standard curves were established by RT‐qPCR analysis of plasmids containing the corresponding BdcRNA cDNAs serially diluted to concentrations ranging from 10^5^–10^10^ ng µL^−1^. The BdcRNA concentration (copies µL^−1^) was calculated based on the amount of cDNA as referred to a standard curve, which corresponded to equal amounts of BdcRNAs extracted from 1 mg mycelia.

### Transcriptome and Proteomic Analysis

Fungal RNA extraction and transcriptome analysis were performed in triplicate by Beijing Novogene Bioinformatics Technology Co. Ltd. according to their pipeline. Briefly, sequencing libraries were generated using the NEBNext Ultra RNA Library Prep Kit for Illumina (NEB, USA) with 1 µg of total RNAs added index codes to attribute sequences to each sample sequenced using an Illumina Novaseq platform and generated 150 bp paired‐end reads. The raw paired‐end reads were trimmed, quality controlled, and aligned to the reference genome using Hisat2 v2.0.5. DEG analysis was performed using the DESeq2 R package (1.16.1).

Fungal protein extraction and proteomic analysis were performed by Jingjie PTM BioLab (Hangzhou) Co. Ltd. Briefly, fungal proteins were extracted, digested with trypsin, dissolved in 0.1% formic acid (solvent A), and directly loaded onto a home‐made reverse‐phase analytical column (15‐cm length, 75 µm i.d.). The peptides were subjected to NSI source followed by tandem mass spectrometry (MS/MS) in Q Exactive Plus (Thermo) coupled online to the UPLC.

GO enrichment analysis of DEG was implemented by the cluster Profiler R package, in which gene length bias was corrected. A *p*‐value < 0.05 was considered to indicate statistically significant enrichment. The GO annotation proteome was derived from the UniProt‐GOA database (http://www.ebi.ac.uk/GOA/). Cluster membership was visualized by a heat map using the “heatmap.2” function from the “gplots” R‐package.

### Statistical Analysis

Descriptive statistics were determined, and chi‐square tests, one‐way ANOVA, and Tukey post‐hoc tests were conducted using SPSS Statistics 17.0 (Win Wrap Basic; http://www.winwrap.com). A *p*‐value ≤ 0.05 was considered to indicate significance. For the growth rate and virulence assays, the mean values for the biological replicates were presented as column charts with error bars representing SD. The graphs were produced in Excel (Microsoft) and GraphPad Prism 7 (GraphPad software).

## Conflict of Interest

The authors declare no conflict of interest.

## Author Contributions

W.X. conceived this study, designed the investigation, wrote the manuscript, and supervised the project. K.D. conducted most of the experiments. C.X. assessed the BdcRNA 1 transfection, monitored growth under different stress conditions, and preformed co‐infection with mycoviruses. R.L. conducted infection of other fungi. J.J. cloned BdRV1 and L.K. participated in some data analysis. S.L., N.H., and G.W. participated in the design of the investigation. I.K.‐L. and R.H.A.C. improved English, presentation, and discussion.

## Supporting information

Supporting Information

## Data Availability

The data that support the findings of this study are openly available in Botryosphaeria dothidea circRNA https://www.ncbi.nlm.nih.gov/nuccore/?term=+Botryosphaeria+dothidea+circRNA, reference number 6.
