# Supplementary material for: Novel Viroid‐Like RNAs Naturally Infect a Filamentous Fungus
Source: Adv Sci (Weinh). 2022 Dec 14;10(3):2204308. doi: 10.1002/advs.202204308 (PMC9875651; doi:10.1002/advs.202204308)
Supplement: Supplementary file 1 — Supporting Information [file ADVS-10-2204308-s001.pdf]

Supplementary figures

Figure S1

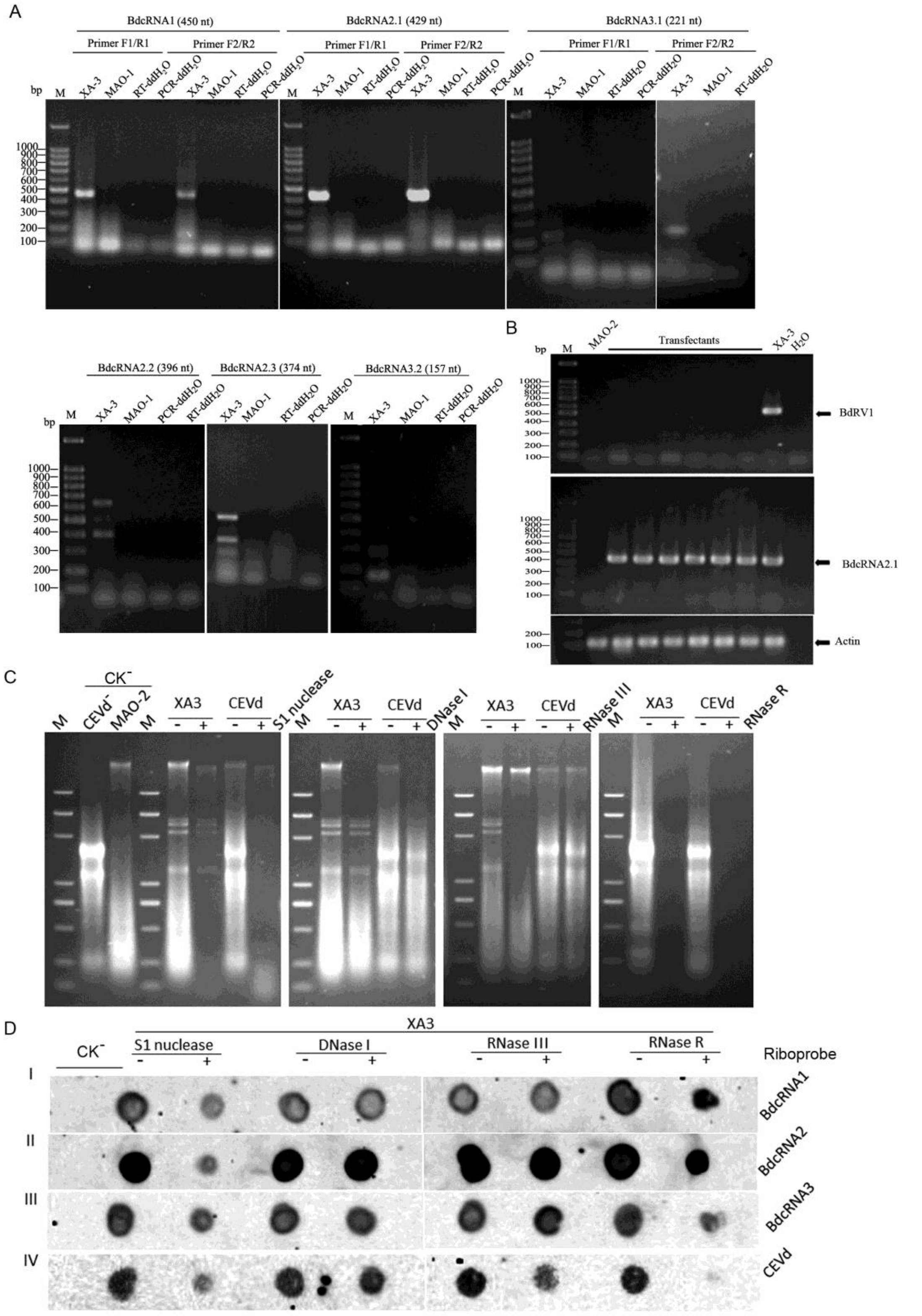

Figure S1. RT-PCR and dot blotting detection of Botryosphaeria dothidea circular RNAs (BdcRNAs) and Botryosphaeria dothidea RNA virus 1 (BdRV1) in strain XA-3,

MAO-2 (or MAO-1), and MAO-2 transfectants of *Botryosphaeria dothidea*. (A) RT-PCR detection of BdcRNAs 1, 2.1, 2.2, 2.3, 3.1 and 3.2 using abutted primer pairs of opposite polarity (-F1 and -R1, Table S1) designed based on obtained contigs by assembling partial cDNAs amplified from each individually purified cRNAs or based on Sanger sequencing (-F2 and -R2, Table S1) in strain XA-3 and MAO-1. (B) RT-PCR identification of BdcRNA2.1 in the protoplast-generating MAO-2 subisolates transfected with BdcRNAs, and BdRV1 and the refer gene *Actin* were involved RT-PCR analysis in parallel to confirm without any contamination of BdRV1 in these subisolates. (C) Electrophoretic analysis on a 1.2% agarose gel of nucleic acid preparation from strain XA-3 before (−) and after (+) digestion with DNase I, S1 nuclease, RNase III and RNase R (I). Nucleic acid preparation from strain MAO-2 and a citrus seedlings free of CEVd were involved as negative controls (CK^-). (D) Dot blotting of the nucleic acid preparations corresponding to those in panel C with the antisense riboprobe of BdcRNA1 (I), 2.1 (II), and 3.1 (III). Nucleic acid preparation of citrus exocortis viroid (CEVd), a circular ssRNA, was included in parallel as control and detected using the CEVd.188 antisense riboprobe (IV). H_2O used for Reverse transcription (RT-H_2O) and PCR (PCR- H_2O) were involved as black controls. M, DNA size marker.

Figure S2

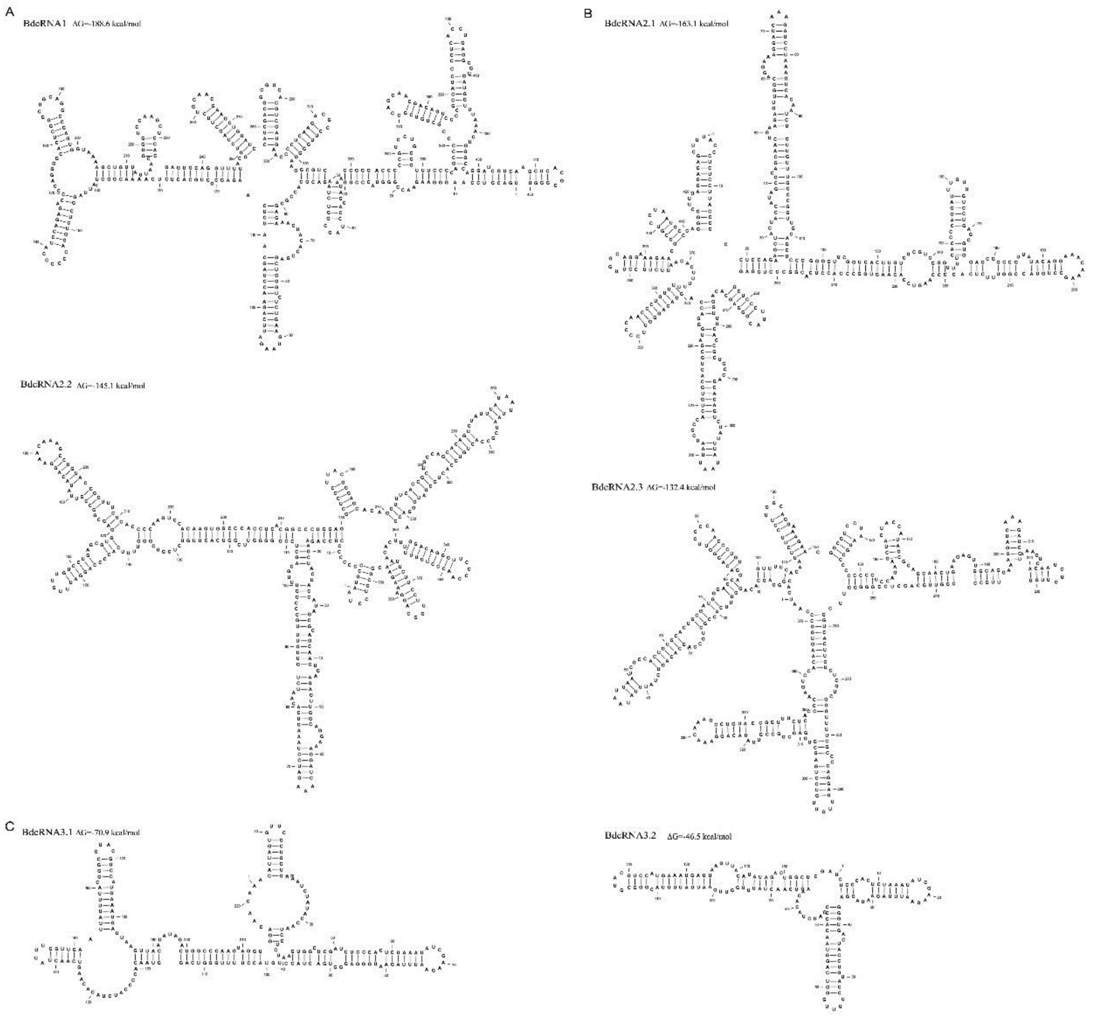

Figure S2. Predicted secondary structures of BdcRNAs in the lowest energy determined using the RNA Structure prediction tool in CLC RNA Workbench software (Version 4.8, CLC bio A/S). (A-C) The secondary structures of BdcRNAs 1 (A), 2.1, 2.2, 2.3 (B), 3.1 and 3.2 (C), respectively.

Figure S3

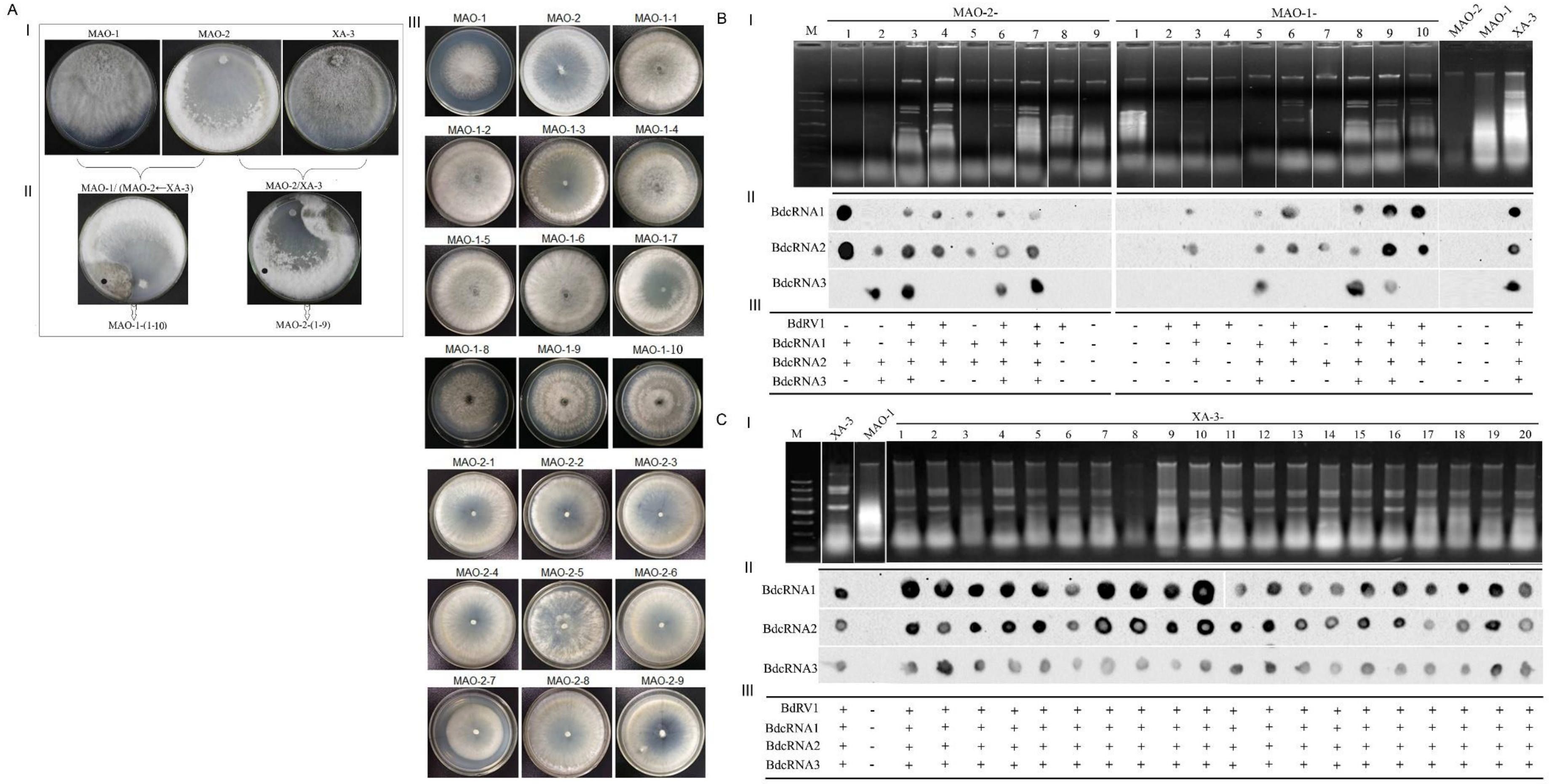

A
I
MAO-1
MAO-2
XA-3
II
MAO-1/ (MAO-2←XA-3)
MAO-2/XA-3
MAO-1-(1-10)
MAO-2-(1-9)
III
MAO-1
MAO-2
MAO-1-1
MAO-1-2
MAO-1-3
MAO-1-4
MAO-1-5
MAO-1-6
MAO-1-7
MAO-1-8
MAO-1-9
MAO-1-10
MAO-2-1
MAO-2-2
MAO-2-3
MAO-2-4
MAO-2-5
MAO-2-6
MAO-2-7
MAO-2-8
MAO-2-9
B
MAO-2-
MAO-1-
M
MAO-2
MAO-1
XA-3
BdcRNA1
BdcRNA2
BdcRNA3
BdRV1
C
XA-3-
M
XA-3
MAO-1
BdcRNA1
BdcRNA2
BdcRNA3
BdRV1

Figure S3 Horizontal and vertical transmission analysis of BdcRNAs and colony appearance of the subisolates. (A) Colony appearance of the *B. dothidea* strains involved in the contact cultures for horizontal transmission. Colonies of strains MAO-1, MAO-2 and XA-3 in single culture (I), contact culture (II), and the subisolates derived from MAO-1 (MAO-1-1 to -7, and MAO-1-1a to 3a) or MAO-2 (MAO-2-1 to -9) after contact culture (III). The "●" indicates the location where a mycelial agar plug was removed for to generate a subisolate derived from strain MAO-1 or MAO-2. (B and C) Nucleic acid preparations (I), dot blotting analysis of BdcRNAs 1 to 3 (II), summary table of the infection of BdcRNAs and BdRV1 (III) of subisolates derived from the horizontal transmission (i.e., from contact cultures) (B) and vertical transmission (i.e., from strain XA-3 conidia) (C), respectively. The "+" and "-" indicate the presence and absence of BdRV1 or BdcRNAs, based on dsRNA detection by 1.2% agarose gel electrophoresis (for BdRV1) and dot blotting (for BdcRNAs), respectively. M, DNA size marker.

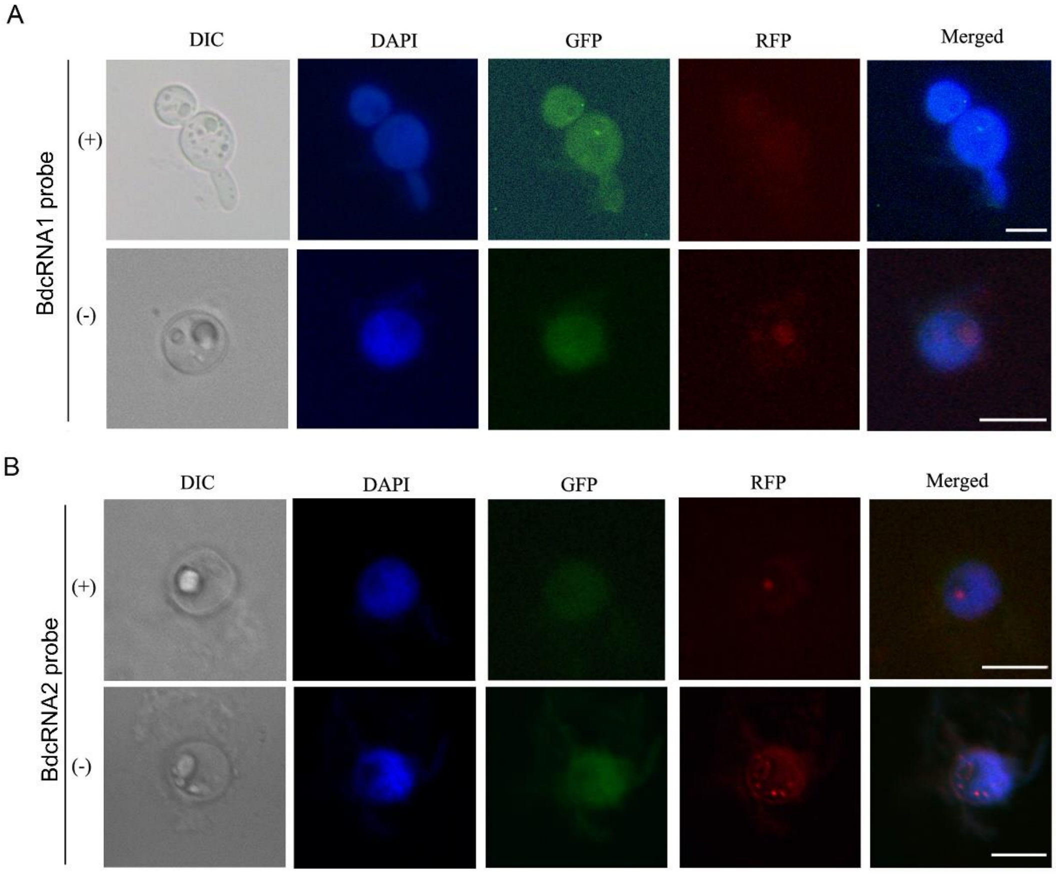

Figure S4. Subcellular location analysis of BdcRNAs. (A and B) FISH to detect subcellular location of plus (+) and minus (-) strands of BdcRNA1 (A) and BdcRNA2.1 (B) in protoplasts of strain XA-3 by Alexa Fluor 488–labeled riboprobes (green fluorescence).The nuclear was indicated with mCherry, binding the fungal nucleosome (red fluorescence), and stained with DAPI in blue color in higher concentration than in cytoplasm.

Figure S5

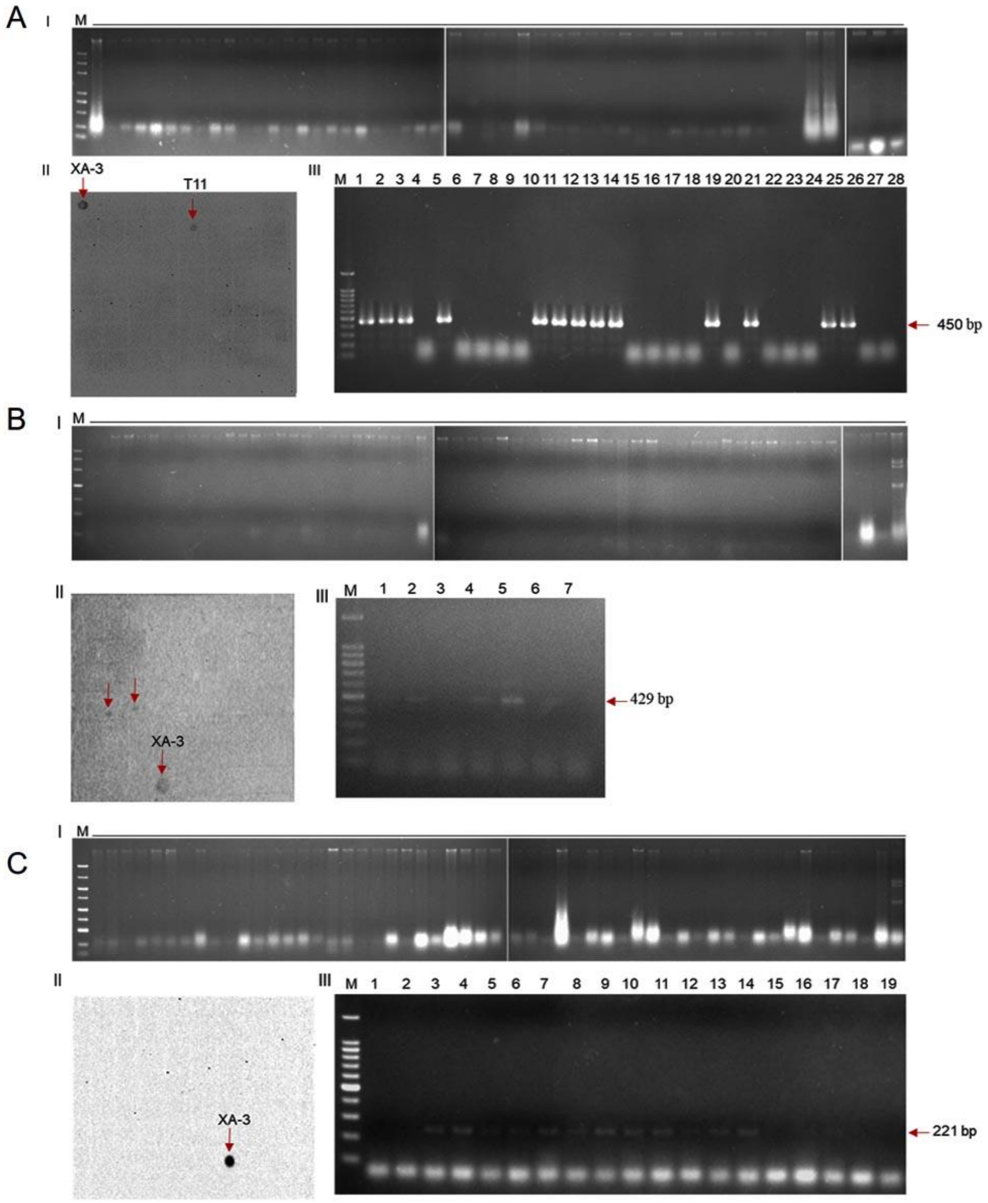

Figure S5. Detection of BdcRNAs in protoplast-generated colonies derived from *B. dothidea* strain MAO-2 transfected with dimeric RNAs that were transcribed from BdcRNA dimeric cDNAs inserted in pGEM-T after digested with *Nde* I. Total 50, 56 and 52 colonies were detected for the transfectants derived from fungal strain transfected with BdCRNAs 1 (A), 2.1 (B) and 3.1 (C), respectively, using dot blotting (II) and RT-PCR (III) based on the extracted nucleic acids using column methods (I). Three positive transfectants of MAO-2 transfected by BdcRNA1 (termed T11), BdcRNA2.1 (T32), and BdcRNA3.1 (T14) were obtained.

Figure S6

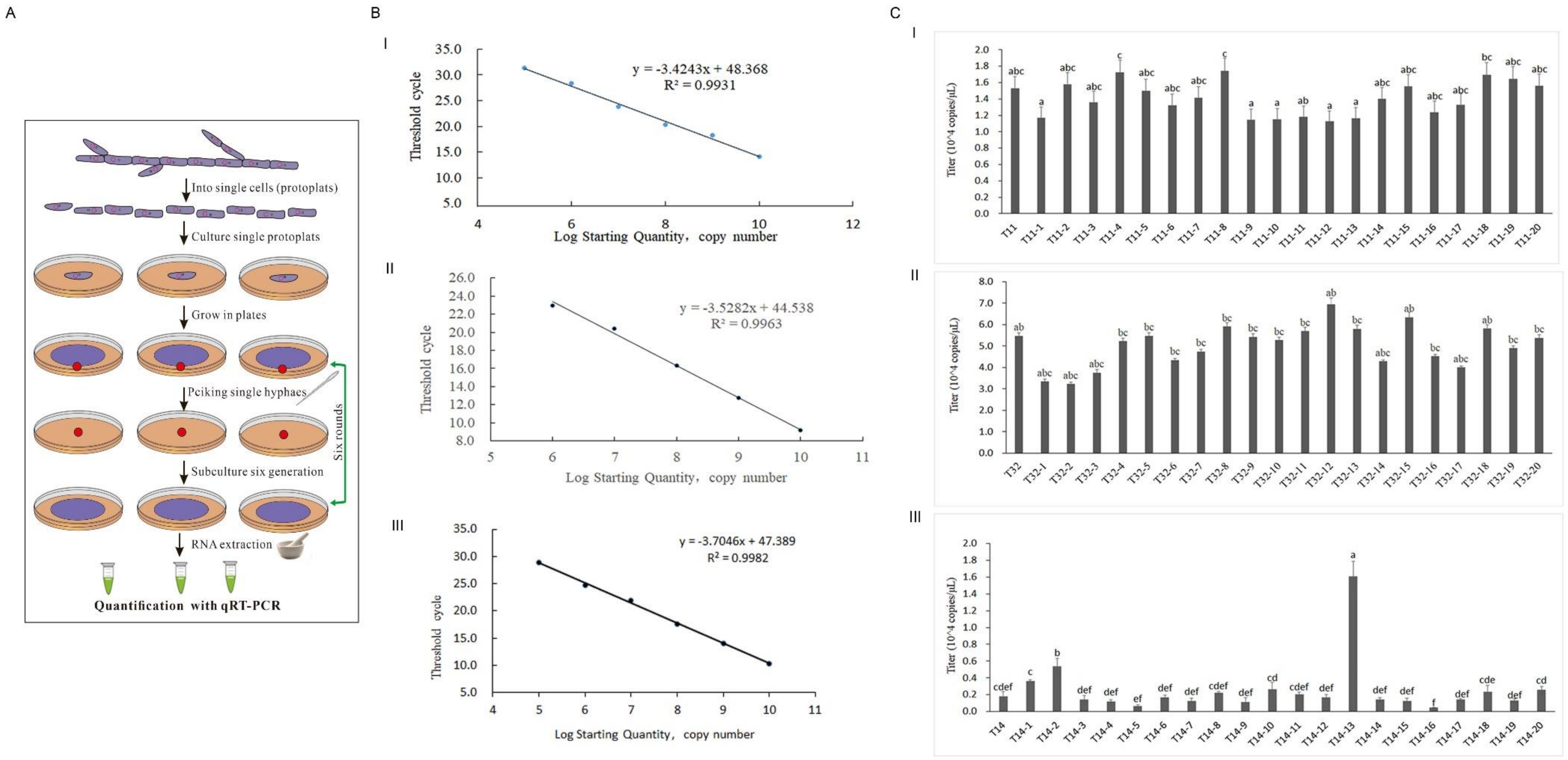

Figure S6 Quantitative analysis of the systematic infection of BdcRNAs in the transfectants of *B. dothidea* strain MAO-2. (A) Flow chart for the

RT-qPCR analysis of the systematic infection and stability of BdcRNAs in individual cells after subculture for six generations. (B) The standard curve for RT-qPCR analysis of cDNA plasmids of BdcRNA1 (I), 2.1 (II) and 3.1 (III) after serially diluted ranging from 10^5 to10^{10} ng/μL. (C) Bar graph for the BdcRNA titers for twenty individual protoplast cells after subculture for six generation for the transfectants of MAO-2 transfected by BdcRNA1 (T11; I), BdcRNA2.1 (T32; II), and BdcRNA3.1 (T14; III). The cell-generated subisolates are serially termed -1 to -20.

Figure S7

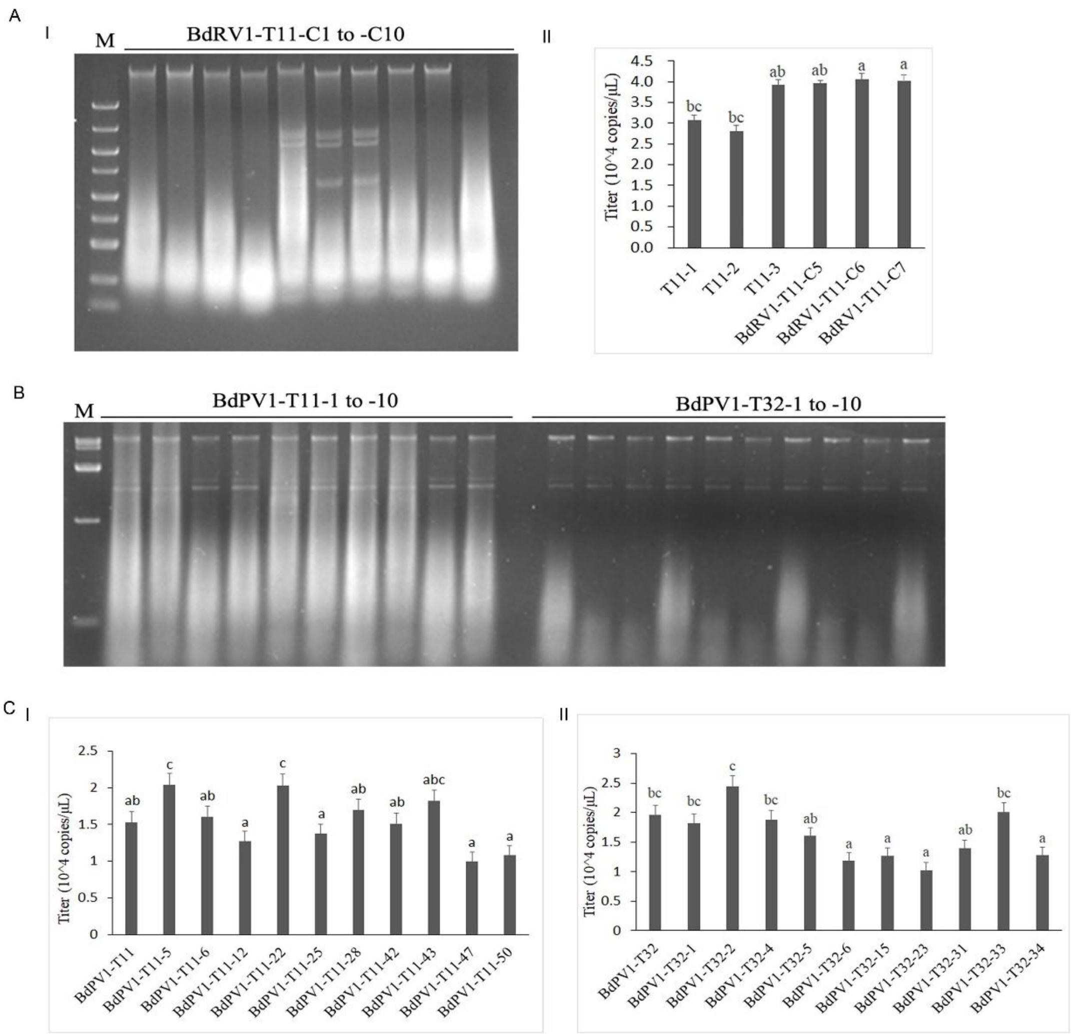

Figure S7 Quantitative analysis of BdcRNA replication affected by mycovirus co-infection in the transfectants T11 and T32 of *B. dothidea* strain MAO-2 after subculture for six generation. (A) Horizontal transmission of BdRV1 into the transfectant T11 (BdcRNA1^{+}, I), and RT-qPCR analysis of BdcRNA1 titer affected by co-infection with BdRV1 (II). Of which, dsRNAs were extracted for BdRV1 detection from ten subisolates (BdRV1-T11-C1 to C10) of T11 after contact culture with MAO-2-9 (containing only BdRV1), indicating subisolate BdRV1-T11-C5 to -C7 infected by BdRV1 (I); and RT-qPCR analysis of BdcRNA1 titers in T11 subisolates absent of BdRV1 (termed T11-1 to -3) and those present of BdRV1 (BdRV1-T11-C5 to C7) (II). (B) Transfection of Botryosphaeria dothidea partitivirus 1 (BdPV1) into T11 (BdcRNA1^{+}) and T32 (BdcRNA2.1^{+}). Of which, dsRNAs were extracted for BdPV1 detection from ten subisolates of T11 and T32 after transfected

by BdPV1 virions. (C) RT-qPCR analysis of BdcRNA1 (I) and BdcRNA2.1 (II) titers in the subisolates of T11 and T32 to analyze the effect by co-infection with BdPV1, respectively. M, DNA size marker.

Figure S8

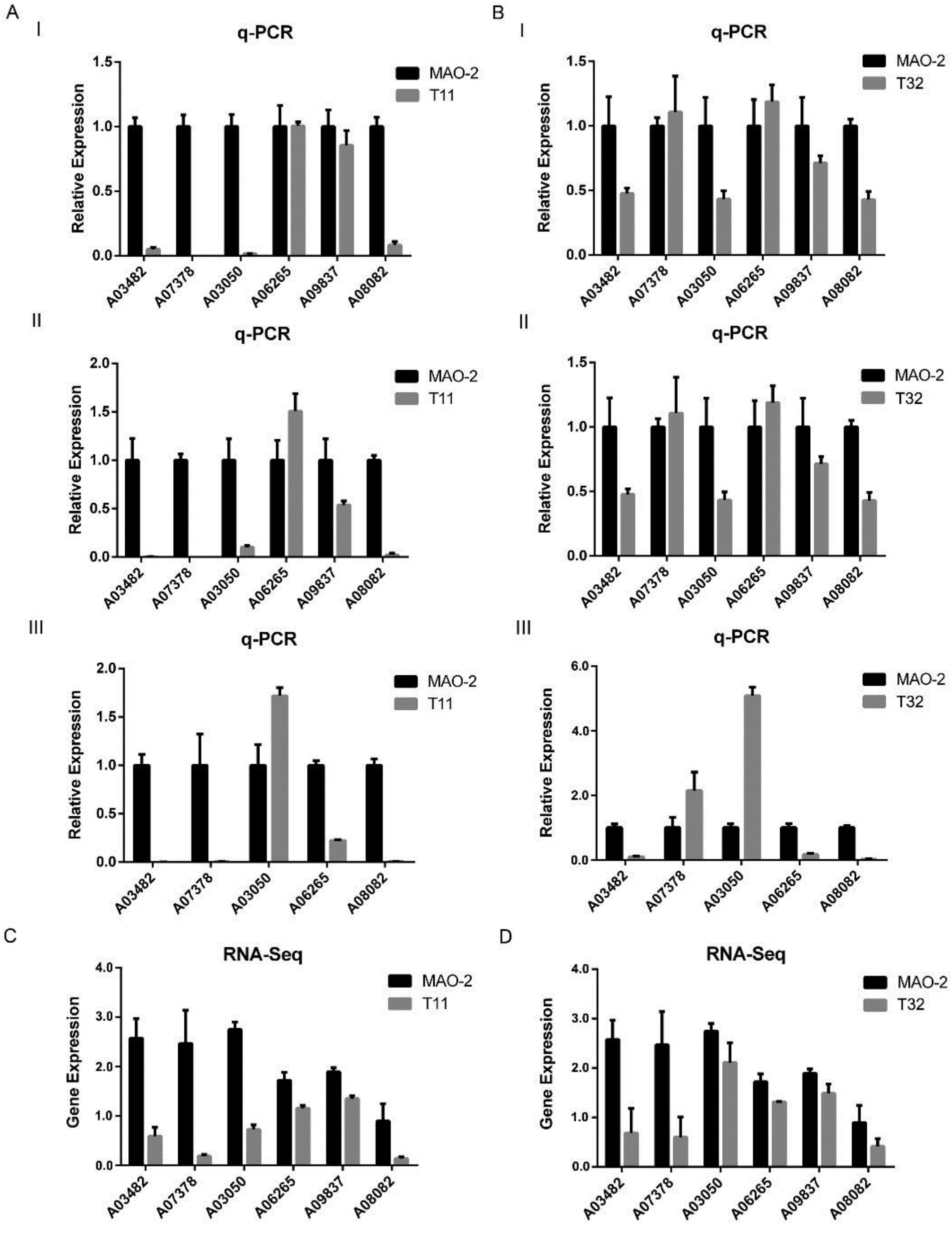

Figure S8 RT-qPCR analysis of the relative expression changes of six genes in strain MAO-2 and the transfectants T11 and T32. RT-qPCR analyses were conducted after cultured at 6 (I), 8 (II) and 10 (III) dpi on PDA, for T11 (A) and T32 transfectants (B) as compared with MAO-2, respectively. The transcriptome sequencing data of mycelia collected at 8 dpi on PDA were involved for comparison for T11 (C) and T32 (D), respectively.

Table S1 The primers used for RT-PCR amplification

Name	Sequences	Product sizes (bp)	Position	Target	Purpose
BdcRNA1-F1	5’-GACGTCCAAGGGAAGAACCG-3’	450	1-21	BdcRNA1	Full-size
BdcRNA1-R1	5’-ACCCGCGTGAGCTTGACGATC-3’		430-450		
BdcRNA1-F2	5’-CACCGCGAGAAACTACACCGA-3’	450	55-75	BdcRNA1	Full-size
BdcRNA1-R2	5’-CGTCTTAGACCGCTAGGTGCT-3’		34-54		
BdcRNA2.1-F1	5’-TACCCTCTCCTTACCCCCCTC-3’	429	1-21	BdcRNA2.1	Full-size
BdcRNA2.1-R1	5’-AAGCTCTCCTAAAGCCCCTGG-3’		409-429		
BdcRNA2.1-F2	5’-GGGTTCGGTCACTTGTCTCGT-3’	429	119-139	BdcRNA2.1	Full-size
BdcRNA2.1-R2	5’-CCGGAGCTGCAACCGGGCAAA-3’		98-118		
BdcRNA2.2-F1	5’-ATGCCCCCCCCCTCCA-3’	396	1-16	BdcRNA2.2	Full-size
BdcRNA2.2-R1	5’-TAGGACGCCGTTCTTTCCTG-3’		377-396		
BdcRNA2.3-F1	5’-AAGAACGGCGTCCTAATGC-3’	375	126-144	BdcRNA2.3	Full-size
BdcRNA2.3-R1	5’-CTGCCAAGGAAGAATTGTGA-3’		104-123		
BdcRNA3.1-F1	5’-AACATTAGTGTTCGCTGGTGA-3’	221	1-21	BdcRNA3.1	Full-size
BdcRNA3.1-R1	5’-TGTTGTCCACCTACTTGGGC-3’		202-221		
BdcRNA3.1-F2	5’-TTATTTTACGGGCGTACGTCCA-3’	221	1573-174	BdcRNA3.1	Full-size
BdcRNA3.1-R2	5’-TTCAACCAAATAGTTGACTTG-3’		137-152		
BdcRNA3.2-F1	5’-TCTCCCACTCTAAATATCG-3’	157	1-19	BdcRNA3.2	Full-size
BdcRNA3.2-R1	5’-TCGAGCCAGTCTATATGTA-3’		139-157		
q450-100-F	5′-TGTTATTAACGTGGTCCAAG-3′	100	208-227	BdcRNA1	Quantitative
q450-100-R	5′-TGAGGGGTTCATCGACGTGA-3′		288-307		
q429-F	5′-GAGTTGGCAGGAAAGGATCAA-3′	94	53-146	BdcRNA2.1	Quantitative
q429-R	5′-AAACCCGACGAGACAAGTGAC-3′				
q221-F	5′-TACCTGTACCTTTTGGGTC-3′	90	94-183	BdcRNA3.1	Quantitative
q221-R	5′-ATCATTTCATGGACGTACGC-3′				
Actin-F	5′-CCGCTCCGTTTCTATGCTCT-3′	114	/	*Actin*	Reference gene
Actin-R	5′-ACCCTCACCGACATACCAGT-3′				

Table S2 Identity and divergence among BdcRNAs.

Identity (%) Divergence	BdcRNA1	BdcRNA2.1	BdcRNA2.2	BdcRNA2.3	BdcRNA3.1	BdcRNA3.2
BdcRNA1		25.6	25.8	32.6	32.5	27.4
BdcRNA2.1	94.5		97.2	66.7	58.6	67.4
BdcRNA2.2	91.5	1.3		66.7	56.9	68.9
BdcRNA2.3	91.6	5.2	0.9		34.0	32.6
BdcRNA3.1	114.3	113.5	125.4	111.5		86.1
BdcRNA3.2	118.2	136.1	138.6	132.3	1.9	
